# Network Pharmacology of the Phytochemical Content of Sunflower Seed (*Helianthus annuus* L.) Extract from LC-MS on Wound-Healing Activity and the In Vitro Wound Scratch Assay

**DOI:** 10.3390/plants15020187

**Published:** 2026-01-07

**Authors:** Juthamat Ratha, Tanit Padumanonda, Chawalit Yongram, Pimolwan Siriparu, Suthida Datham, Muhammad Subhan, Chatchavarn Chenboonthai, Ploenthip Puthongking

**Affiliations:** 1 Melatonin Research Group, Khon Kaen University, Khon Kaen 40002, Thailand; juthra@kku.ac.th; 2 Division of Pharmacognosy and Toxicology, Faculty of Pharmaceutical Sciences, Khon Kaen University, Khon Kaen 40002, Thailand; tanpad@kku.ac.th; 3 Radiation Dose Assessment Section, Regulatory Technical Support Division, Office of Atoms for Peace, Bangkok 10900, Thailand; chawalit.y@oap.go.th; 4 Faculty of Pharmaceutical Sciences, Graduate School, Khon Kaen University, Khon Kaen 40002, Thailand; pimolwan.s@kkumail.com (P.S.); suthida.tham@kkumail.com (S.D.); muhammad.s@kkumail.com (M.S.); 5 General Drugs House Co., Ltd., 90, 90/1 Moo 4, Bueng Kham Phroi District, Lam Luk Ka 12150, Thailand; chatchvarn@yahoo.com; 6 Division of Pharmaceutical Chemistry, Faculty of Pharmaceutical Sciences, Khon Kaen University, Khon Kaen 40002, Thailand

**Keywords:** cell migration, immortalised human keratinocyte (HaCaT), molecular docking, chlorogenic acid, indolamine

## Abstract

Sunflower seeds have been reported to be a healthy natural source of polyphenols. This study aimed to explore the mechanisms of potential compounds in sunflower seed extract involved in wound healing; major compounds were investigated through network pharmacology and molecular docking. In an in vitro wound-healing assay applied using an immortalised human keratinocyte (HaCaT) cell model, 10 µg/mL of the sunflower seed extract promoted cell migration in HaCaT cells and led to complete wound closure after 24 h; at a 1 µg/mL concentration, it led to complete wound closure after 72 h. The sunflower seed extract presented moderate-to-strong antioxidant activity. Liquid chromatography–mass spectrometry and high-performance liquid chromatography were used to identify the major compounds present in the sunflower seed extract. Forty-seven compounds were identified, among which chlorogenic acid was the most abundant phenolic compound. Network pharmacology was used to identify wound-healing-related targets. In total, 252 proteins were linked to the 47 compounds. Cyto-Hubba analysis identified 10 hub proteins with a strong correlation with wound healing. Molecular docking was used to assess the ability of the major compounds in the sunflower seed extract to combat NF-κB1, EGFR, and MMP9. Chlorogenic acid showed higher binding affinity to all targets. Moreover, its pharmacokinetic properties were well distributed in the plasma (VDss = 0.377 log L/kg), and they were not a carcinogen and did not cause skin sensitisation. In conclusion, the findings suggest that the sunflower seed extract is a potential source of bioactive compounds that can enhance wound healing and can be developed to create a transdermal application.

## 1. Introduction

Wound healing is a natural biological response that aims to restore the morphology and function of the skin after it has been damaged [1,2]. This complicated process involves tissue connections and a myriad of growth factors [3]. Normal wound healing is characterised by the orderly and contiguous stages of haemostasis, inflammation, proliferation, and remodelling [4]. Identifying a natural product or drug that could contribute to all wound-healing phases would be ideal, especially if it is low-cost and has minimal toxicity.

Sunflowers (*Helianthus annuus* L.) are cultivated around the world and have edible seeds that are a rich source of healthy unsaturated fats, proteins, fibres, flavonoids, phenolic acids, and coumarins [5,6]. Sunflower seeds are well known as a source of vegetable oils, including linoleic acid, oleic acid, and palmitic acid [7]. These seeds contain several natural polyphenols, including chlorogenic acid, caffeic acid, gallic acid, *p*-coumaric acid, quercetin, and kaempferol, which have potential health benefits [8,9,10,11]. These natural polyphenols could act as antioxidants to ameliorate oxidative stress and enhance wound healing [12,13]. Chlorogenic acid is one of the most notable phenolic acids in sunflower seeds, with potential therapeutic value for wound healing [14,15,16]. Gai et al. [9] identified the presence of phenolic compounds through methanol extraction of sunflowers at the mid-flowering stage and found that these plants are a good source of antioxidant activity. Extraction under polar conditions could increase the value of this plant as an alternative source of health-promoting elements. Previous investigations studied the effect of sunflower seed oil on wound-healing activity in vivo [17,18], but no studies have investigated the effects of phytochemicals from sunflower seed extract on wound healing and the mechanisms of action involved.

At present, plants are demonstrating increasing beneficial effects in human healthcare, therapeutics, and disease prevention, related to the presence of polyphenols, such as phenolic acids, flavonoids, and coumarins [19,20]. The demand for natural ingredients has led to increasing interest in these elements’ use in skin products and healthcare, as well as skin wound healing [20]. The objectives of this study were to explore the wound-healing activity of sunflower seed extract in an in vitro wound-healing model using an immortalised human keratinocyte cell line (HaCaT), as well as to assess the phytochemical profiling of the sunflower seed extract and examine its antioxidant activity. Consequently, this study determined how the major components of sunflower seed extract contribute to wound healing at the cellular and molecular levels using network pharmacology and molecular docking, respectively. Our findings could inspire the development of sunflower seed extract as a potential natural product for use in wound-healing products.

## 2. Results

### 2.1. Antioxidant Activity of the Sunflower Seed Extract

The methanolic sunflower seed extract was assessed for antioxidant activity based on its ability to scavenge 2,2-diphenyl-2-picrylhydrazyl (DPPH) and 2,2′-azino-bis(3-ethylbenzthiazoline-6-sulphonic acid) (ABTS) radicals and its ferric-reducing antioxidant power (FRAP). For the DPPH assay, the half maximal inhibitory concentration (IC_50_) was 54.01 ± 0.81 µg/mL, which indicates strong activity according to the classification method of Kusumawati et al. [21]. Moreover, the IC_50_ for the positive control Trolox was 7.09 ± 0.00 µg/mL, indicating that it has very strong antioxidant power. For the ABTS assay, IC_50_ was 114.75 ± 0.75 µg/mL, classifying the sunflower seed extract as having moderate antioxidant activity. Trolox was 6.05 ± 0.11 µg/mL, which classifies it as having strong antioxidant activity (Figure S1). In addition, the sunflower seed extract had an FRAP of 2.08 ± 0.04 mmole Fe^2+^/g·DW, lower than that of Trolox (24.38 ± 0.78 mmole Fe^2+^/g).

### 2.2. Wound-Healing Assay

**Figure 1:**
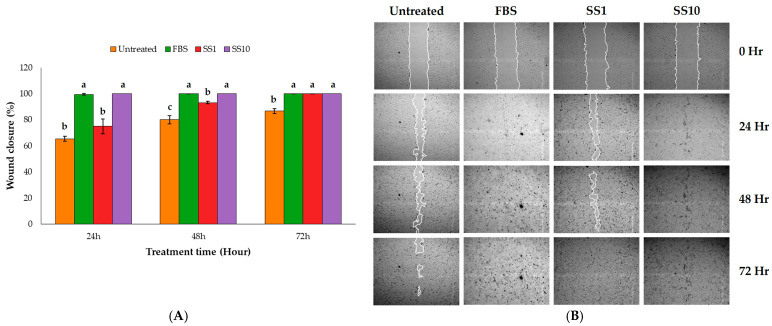
The effect of the sunflower seed (SS) extract on in vitro wound healing using a scratch assay with HaCaT cells. (**A**) The graph shows the percentage wound closure at 24, 48, and 72 h for various treatments: the untreated control, 10% foetal bovine serum (FBS, the positive control), and two SS extract concentrations (1 and 10 µg/mL, SS1 and SS10, respectively). For each time, the columns with a different lowercase letter (a–c) differ significantly (Turkey’s test, n = 3, *p* < 0.05). (**B**) Representative images of the HaCaT cells at various times while conducting the wound-healing assay.

This study evaluated the wound-healing activity of the sunflower seed extract using a scratch assay with keratinocyte HaCaT cells. First, a 3-(4,5-dimethylthiazol-2-yl)-2,5-di-phenyltetrazolium bromide (MTT) assay was performed, according to ISO 10993-5 [22], to determine the effects of different concentrations of the sunflower seed extract on HaCaT viability. The tested concentrations (10–400 µg/mL) did not affect cell viability. Extract concentrations of 1 and 10 µg/mL were chosen to measure the wound scratch assay. After treating the cells, wound closure was evaluated at 24, 48, and 72 h. After treatment with 1 µg/mL of sunflower seed extract, the percentage of closure was 75.04% ± 5.74% at 24 h, and complete closure occurred (100%) at 72 h. When treated with 10 µg/mL, complete wound closure occurred after 24 h, similarly to the cells treated with the positive control, that is, a medium containing 10% foetal bovine serum (FBS; Figure 1A,B). Of note, all treated groups showed a significantly higher migration rate than the untreated group. The faster wound closure noted at the higher sunflower seed extract concentration may have been due to the higher concentrations of some natural polyphenols contained in the extract. Therefore, phytochemical profiling of the sunflower seed extract was further carried out.

### 2.3. Phytochemical Profiling

#### 2.3.1. Analysis of the Sunflower Seed Extract via Liquid Chromatography–Mass Spectrometry (LC-MS)

The LC-MS chromatograms of the sunflower seed extract, obtained in both positive and negative ionisation modes, are presented in Figure S2 of the Supplementary Materials (Supplementary Materials S1—Figures and Tables). A total of 4236 and 15,030 compounds were detected in negative and positive ionization modes, respectively (Supplementary Materials S2—Raw data of LCMS). The raw LC-MS data were processed with a data-filtration based on a mass error ≤ ±20 ppm and an identification score of ≥0.70 [23,24], which indicate reliable LC-MS results. The sunflower seed extract was identified as containing 48 compounds: 25 compounds in the negative mode and 23 in the positive mode. In the negative mode, the most abundant compound was D-(-)-Quinic acid, followed by chlorogenic acid and 3,4-di-*O*-caffeoylquinic acid. Other identified compounds included phenolic acids and their derivatives (e.g., caffeic acid and berginin); the flavonoids eriodictyol, tricin-5-glucoside, isookanin-7-*O*-glucoside, and plantaginin; and low quantities of linoelaidic acid and α-linolenic acid, which are well known to be present in sunflower seed oil. Tryptophan, an essential amino acid, was also identified in the extract, but in a relatively low quantity (Table 1 and Table S1).

In the positive mode, the most abundant compound was 7,8-dimethoxycoumarin, followed by chlorogenic acid and caffeoylcholine. The presence of quinic acid and coumaric acid derivatives, including 4-coumaroylcholine, 3,4-di-*O*-caffeoylquinic acid, and skimmin, as well as a low amount of *p*-coumaric acid, was also noted in this study. The amino acids arginine, isoleucine, valine, and tyrosine were identified (Table 2 and Table S2).

#### 2.3.2. Quantification of Phenolic and Indolamine Compounds

The sunflower seed extract had a total phenolic content (TPC) of 44.61 ± 1.51 mg gallic acid equivalents (GAEs)/g dry weight (DW) and a total flavonoid content (TFC) of 2.30 ± 0.04 mg quercetin equivalents (QUEs)/g·DW. Through LC-MS analysis, phenolic acids such as chlorogenic acid, caffeic acid, and *p*-coumaric acid were identified; therefore, quantitative analysis through high-performance liquid chromatography (HPLC) was subsequently performed. Quantitative analysis was conducted on well-known phenolic compounds found in plants, including gallic acid, protocatechuic acid, *p*-hydroxybenzoic acid, caffeic acid, syringic acid, chlorogenic acid, *p*-coumaric acid, ferulic acid, sinapic acid, rutin, and quercetin. Among these 11 phenolic compounds, the contents of chlorogenic acid and caffeic acid were 38 ± 0.02 mg/g·DW and 5.17 ± 0.06 µg/g·DW. *p*-Coumaric acid was detected at trace levels and could not be quantified. Phenolic acids were validated using HPLC-DAD in our previous publication [25]. The limit-of-detection (LOD) and limit-of-quantification (LOQ) values of chlorogenic acid were 0.025 µg/mL and 0.1 µg/mL, respectively, whereas the LOD and LOQ values of both caffeic acid and *p*-coumaric acid were 0.005 µg/mL and 0.01 µg/mL, respectively. The sunflower seed extract also contained tryptophan, an essential amino acid, and two products that can be derived from it, namely serotonin and melatonin. This study found a tryptophan content of 15.52 ± 0.04 µg/g·DW and a melatonin content of 0.02 ± 0.00 µg/g·DW; however, this study did not detect serotonin (Table 3 and Figures S3 and S4). As indolamines were analysed via HPLC-FLD, the LOD and LOQ values of tryptophan were 0.01 µg/mL and 0.05 µg/mL, respectively, while the LOD values of melatonin and serotonin were both 0.0025 µg/mL, and the LOQ values of melatonin and serotonin were both 0.05 µg/mL.

### 2.4. Network Pharmacology Was Used to Determine the Wound-Healing Effect of the Sunflower Seed Extract

#### 2.4.1. Screening of Wound-Healing-Associated Gene Targets

**Figure 2:**
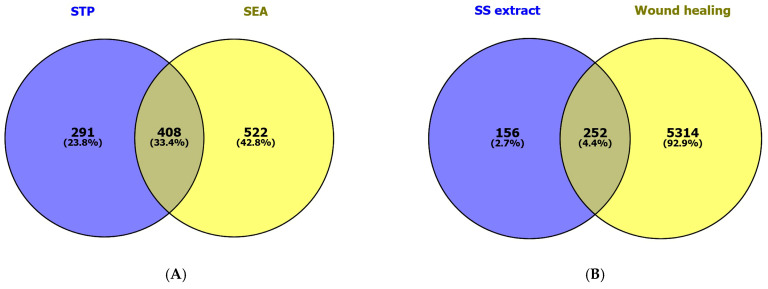
The Venn diagrams show (**A**) the common targets of compounds found in sunflower seed (SS) extract based on searches of the SwissTargetPrediction (STP) and Similarity Ensemble Approach (SEA) databases, as well as (**B**) the common targets from the SS extract and therapeutic targets associated with wound healing.

Our network pharmacology analysis revealed 47 compounds (without duplicates) associated with 699 and 930 targets, respectively, based on searches of the SwissTargetPrediction and Similarity Ensemble Approach databases. The Venn diagram in Figure 2A shows 408 targets (33.4%) in common. A total of 5566 therapeutic targets from the GeneCards database were linked with the wound-healing process (using “wound healing” as a keyword). Comparing the 408 targets related to the sunflower seed extract and the 5566 therapeutic targets revealed 252 (4.4%) common targets directly related to wound-healing (Figure 2B).

#### 2.4.2. Protein–Protein Interactions (PPIs)

**Figure 3:**
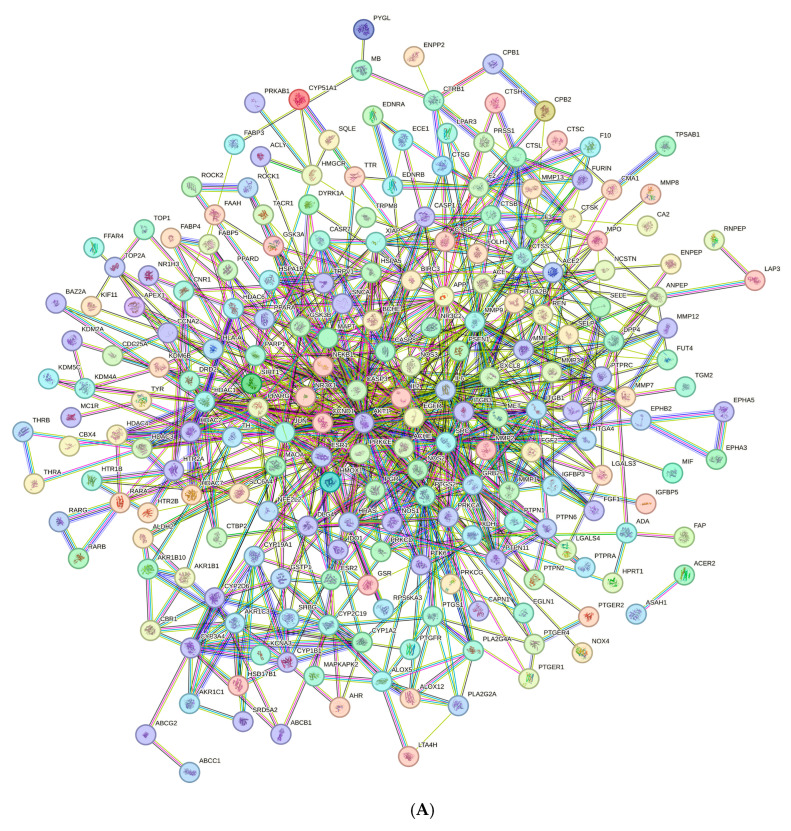

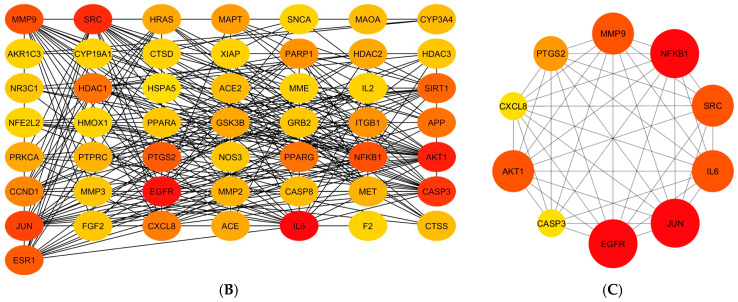
Protein–protein interaction networks: (**A**) The original network exported from the STRING database, and networks with (**B**) 50 targets and (**C**) 10 hub targets involved in the wound-healing activity of the sunflower seed extract. Each node represents a gene, and the larger and darker nodes represent higher degree scores. Cytoscape software (version 3.10.4) was used for visualisation.

The PPI network was constructed using the STRING database based on the 252 common target proteins. It comprised 251 nodes (i.e., the target proteins) connected with 827 edges (i.e., the interactions between the proteins); only HSPA1B had no interactions (Figure 3A). Nodes represented target proteins, edges represented the interactions between proteins, and lines represented the relationships between target proteins. The average node degree was 6.59 and the average local clustering coefficient was 0.436. The PPI enrichment *p*-value was <1 × 10^−16^, and the network had significantly more interactions (827 edges) than expected (279 edges).

The CytoHubba plugin was used to represent interactions between the potential targets with a degree value >10 (the redder the nodes, the higher the degree; the yellower nodes, the lower the degree). Ten hub targets were used to construct a network based on fifty targets (Figure 3B) and screened using the degree, betweenness, and closeness methods (Figure 3C). The 10 hub targets were matrix metalloproteinase 9 (MMP9), nuclear factor kappa B subunit 1 (NF-κB1), proto-oncogene tyrosine-protein kinase Src (SRC), interleukin 6 (IL-6), transcription factor AP-1 (JUN), epidermal growth factor receptor (EGFR), caspase-3 (CASP3), RAC-alpha serine/threonine-protein kinase (AKT1), interleukin 8 (CXCL8), and prostaglandin-endoperoxide synthase 2 (PTGS2). Among these hub targets, EGFR, JUN, and NF-κB1 were derived from the group with the highest degree value, followed by the group containing MMP9, SRC, IL6, and AKT1; the group containing PTGS2; and, finally, the group containing CXCL8 and CASP3 (Figure 3C).

#### 2.4.3. Gene Ontology (GO) and Kyoto Encyclopaedia of Genes and Genomes (KEGG) Pathway Analyses

**Figure 4:**
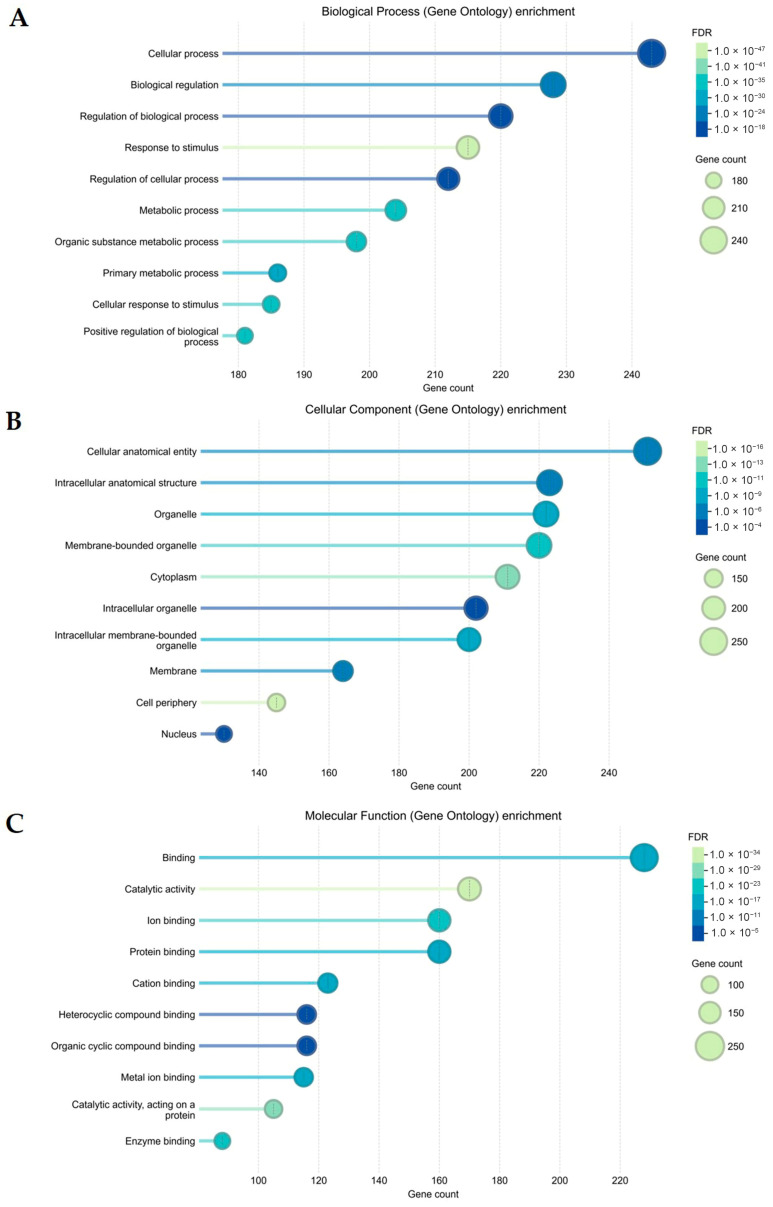
Gene Ontology (GO) enrichment analysis of potential targets of the sunflower seed extract contributing to its wound-healing activity. The top 10 GO functional terms are categorised as (**A**) biological process, (**B**) cellular component, and (**C**) molecular function. The size of the dot indicates the number of genes. The colour of the dot represents a false discovery rate (FDR) value (*p* < 0.01).

The 252 overlapping targets were uploaded to the STRING database to determine the functional mechanisms and pathways of the sunflower seed extract that influence wound-healing activity. GO analyses were ranked according to the false discovery rate (FDR) value (*p* < 0.01): 1654 for biological process, 202 for cellular component, and 121 for molecular function. These cellular processes, metabolic processes, biological regulations, and responses to stimulus (Figure 4A) are mainly mediated in the organelles, cytoplasm, membrane, cell periphery, and nucleus (Figure 4B). The targets are mainly involved in binding ions and cations, proteins, and organic cyclic compounds, as well as in catalytic binding (Figure 4C).

KEGG pathway analysis revealed 177 significantly enriched pathways, with the top 10 pathways being consistent with GO enrichment analysis. The targets mainly regulate pathways in cancer, microRNAs in cancer, neuroactive ligand–receptor interaction, proteoglycans in cancer, serotonergic synapse, human cytomegalovirus infection, apoptosis, the AGE–RAGE signalling pathway in diabetic complications, focal adhesion, and the relaxin signalling pathway. The most relevant pathway via which the sunflower seed extract contributes to wound healing might involve focal adhesions, which are related to cellular migration.

**Figure 5:**
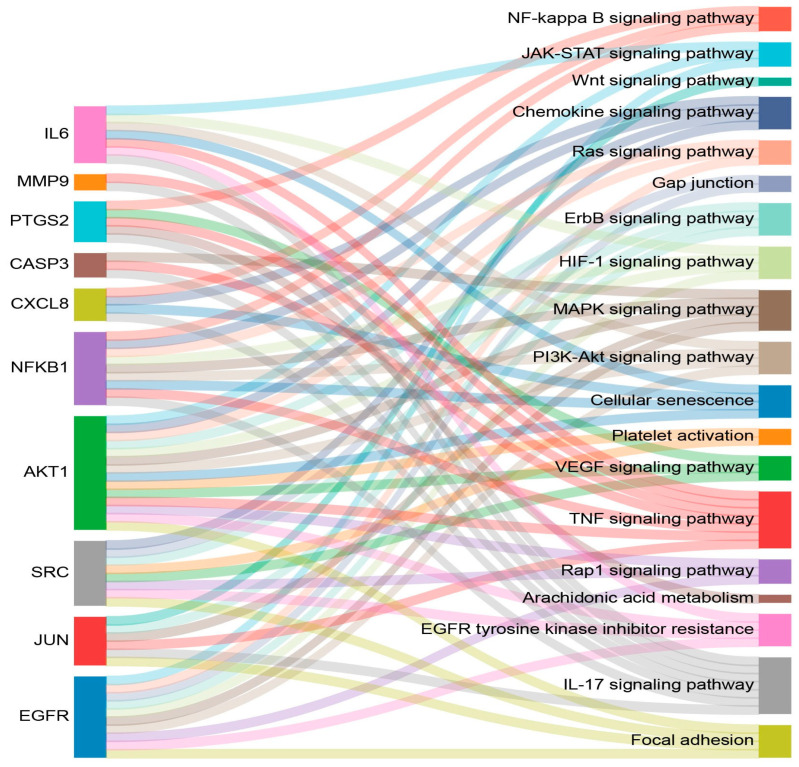
The Sankey diagram displays the relationships between the 10 hub targets and the 19 enriched wound-healing-related pathways. The diagram was generated using SRplot software (https://www.bioinformatics.com.cn/en, accessed on 23 June 2025).

Based on the significantly enriched KEGG pathways, 19 pathways related to the wound-healing process were identified; the target genes are listed in Table 4. Wound healing involves a cascade of haemostasis, inflammation, proliferation, and remodelling. The pathways involved in haemostasis include the HIF signalling pathway, the JAK–STAT signalling pathway, platelet activation, gap junctions, and EGFR tyrosine kinase inhibitor resistance. The pathways related to inflammation include the IL-17 signalling pathway, the TNF signalling pathway, the chemokine signalling pathway, the NF-kappa B signalling pathway, arachidonic acid metabolism, the MAPK signalling pathway, and cellular senescence. The pathways associated with proliferation include focal adhesion, the Rap1 signalling pathway, the PI3K–Akt signalling pathway, the Ras signalling pathway, and the Wnt signalling pathway. The VEGF signalling pathway is related to remodelling. The relationships between the 10 hub targets and the 19 pathways related to wound healing are illustrated in a Sankey diagram (Figure 5).

### 2.5. Molecular Docking

Based on the results of the network pharmacology analysis, NF-κB (PDB ID: 4KIK), EGFR (PDB ID: 1M17), and MMP9 (PDB ID: 1GKC) were identified as the targets with the highest degree scores in this study; they are involved in the inflammation, proliferation, and remodelling phases of wound healing, respectively. Twenty-one compounds with a relative intensity >1% from both the negative and positive modes were selected to examine molecular docking with the three aforementioned hub targets. In silico molecular docking measures the binding free energy (ΔG) of a ligand to a protein, with a large negative value indicating a strong interaction. This analysis is also used to predict possible interactions between a ligand and a protein, including hydrogen bonding, which is crucial for interactions.

For the inflammation target (NF-κB), the standard ligand KSA shows the highest ΔG (−11.90 kcal/mol). The ΔG of the compounds in the sunflower seed extract range from −3.30 to −10.32 kcal/mol. The top 5 compounds with the highest binding affinity are tricin 5-glucoside (compound **6**, −10.32 kcal/mol), isookanin-7-*O*-glucoside (compound **8**, −10.06 kcal/mol), 3,4-di-*O*-caffeoylquinic acid (compound **7**, −9.16 kcal/mol), eriodictyol (compound **19**, −8.71 kcal/mol), and chlorogenic acid (compound **16**, −8.12 kcal/mol). Ligand KSA forms hydrogen bonds with GLU97, GLU149, and CYS99, as well as pi–sulphur and pi–alkyl interactions. All compounds form hydrogen bonds with amino acids at the active site of NF-κB.

For the proliferation target (EGFR), the target compounds in the sunflower seed extract present a ΔG ranging from −2.76 to −7.12 kcal/mol. Two compounds show the best binding, namely eriodictyol (compound **19**, −7.12 kcal/mol) and cimifugin 4′-*O*-beta-D-glucopyranoside (compound **20**, −7.12 kcal/mol), followed by the standard ligand AQ4 (−7.00 kcal/mol), tricin 5-glucoside (compound **6**, −6.93 kcal/mol), isookanin-7-*O*-glucoside (compound **8**, −6.82 kcal/mol), and chlorogenic acid (compound **16**, −6.73 kcal/mol). AQ4 forms two conventional hydrogen bonds with MET769 and CYS773 and six pi–alkyl/alkyl interactions. Eriodictyol, cimifugin 4′-*O*-beta-D-glucopyranoside, chlorogenic acid, and isookanin-7-O-glucoside also form hydrogen bonds with MET769 and CYS773.

Finally, for the remodelling target (MMP9), ΔG ranges from −3.81 to −9.41 kcal/mol. Among the tested compounds, cimifugin 4′-*O*-beta-D-glucopyranoside has the lowest ΔG (compound **20**, −9.14 kcal/mol), indicating better binding than the standard ligand NFH (−7.87 kcal/mol). The other top five targets are chlorogenic acid (compound **16**, −8.90 kcal/mol), isookanin-7-*O*-glucoside (compound **8**, −8.65 kcal/mol), eriodictyol (compound **19**, −8.29 kcal/mol), and 4-coumaroylcholine (compound **18**, −8.21 kcal/mol). NFH forms hydrogen bonds with the MMP9 residues LEU188, ALA189, HIS401, HIS405, HIS411, and TYR423. The top five compounds also form hydrogen bonds with LEU188 and ALA189 (Table 5 and Figures S5–S7). Table S3 also shows the binding interactions of these compounds.

Based on the molecular docking results, isookanin-7-*O*-glucoside (compound **8**), eriodictyol (compound **19**), and chlorogenic acid (compound **16**) show the strongest binding to NF-κB, EGFR, and MMP9, respectively.

### 2.6. Pharmacokinetic Properties

This study used the pkCSM online tool to predict the following pharmacokinetic properties: absorption (A), distribution (D), metabolism (M), excretion (E), and toxicity (T). We assessed transdermal absorption based on water solubility, Caco-2 permeability, and skin permeability; distribution based on the volume of distribution (VDss) and the fraction unbound; metabolism based on the ability to inhibit cytochrome P450 (CYP) isoforms (CYP1A2, CYP2C19, CYP2C9, CYP2D6, and CYP3A4) and serve as a substrate for CYP2D6/CYP3A4; excretion based on total clearance; and toxicity based on the Ames test, the maximum tolerated dose, and skin sensitisation.

All compounds show moderate solubility, poor Caco-2 permeability, and reasonable skin penetration based on the pkCSM criteria [26]. In terms of distribution, most compounds show low VDss values (log VDss < 0.45), indicating a greater distribution in plasma than tissue; the exceptions are compounds **7**, **14**, **17**, and **18**. All compounds show a very low fraction unbound in the plasma (0.054–0.914 Fu). Most compounds do not inhibit CYPs inhibitors, except for compounds **9**, **13**, **14**, **15**, and **18**, which are predicted to inhibit CYP1A2. None of the compounds are CYP substrates, except for compound **9** (predicted to be a CYP3A4 substrate). Regarding excretion, all compounds except for compound **8** have high total clearance, indicating fast removal from the blood or plasma. In terms of toxicity, only compounds **6**, **8**, **14**, **15**, and **19** are predicted to be carcinogenic based on the Ames test. Most compounds have a predicted maximum tolerated dose above 0.477 log mg/kg/day (the maximum tolerated dose in humans). Finally, none of the compounds cause skin sensitisation (Table S4).

Based on our analysis, chlorogenic acid (compound **16**) may be the main constituent of sunflower seed extract responsible for its wound-healing activity. It may promote wound healing by binding to NF-κB, EGFR, and MMP9 during the inflammation, proliferation, and remodelling phases, respectively. Chlorogenic acid shows high binding affinity for the targets and good pharmacokinetic properties compared with the compounds with the highest ΔG values (compounds **8** and **19**).

## 3. Discussion

Sunflower seeds are a nutritious source of phenolic acids, flavonoids, and fatty acids. They are used commercially as snacks, baking ingredients, and for oil production. Several compounds in sunflower seeds have health benefits, including antioxidant [27,28], antimicrobial [29], anti-diabetic [30], and wound-healing [17,18] activities. This study examined the wound-healing activity of sunflower seed extract. In a wound-healing assay involving HaCaT cells, the sunflower seed extract had a dose-dependent effect on cell migration (Figure 1). The higher concentration of 10 µg/mL led to complete wound closure after 24 h. Based on these results, in this study, the compounds that exert this wound-healing activity in the sunflower seed extract were investigated.

The sunflower seed extract had a strong DPPH radical scavenging ability, with an IC_50_ (51.04 µg/mL) 19–25-fold higher than that in a previous report (0.99–1.29 mg/mL) [28]. It had a moderate ability to scavenge ABTS radicals (IC_50_ = 114.75 µg/mL), similarly to the study by Hwang et al. [27] (IC_50_ = 174.65 µg/mL). The FRAP of the sunflower seed extract (2.08 mmole Fe^2+^/g·DW) is within the range of 0.25–68.6 µmole Fe^2+^/g·DW reported previously [9,31]. The TPC of 44.61 mg GAE/g·DW is 5–20-times higher than that reported in other sunflower seed studies (2.73–8.31 mg GAE/g·DW) [7,32]. The high TPC is consistent with the findings of Amakura et al. [33], who stated that phenolic compounds are a major source of antioxidant power. The TFC of the sunflower seed extract (2.8 mg QUE/g·DW) is lower than the value of 23.57 mg QUE/g·DW reported by Gagour et al. [34].

Phytochemical profiling using LC-MS identified D-(-)-quinic acid, 7,8-dimethoxycoumarin, chlorogenic acid, 3,4-di-*O*-caffeoylquinic acid, caffeoylcholine, and other phenolics and flavonoids such as caffeic acid, *p*-coumaric acid, berginin, and eriodictyol. Moreover, Amakura et al. [33] and Pedrosa et al. [35] both identified chlorogenic acid and caffeic acid in sunflower seeds; Gai et al. [9] identified chlorogenic acid, 3,4-di-*O*-monocaffeoylquinic acid, and 4,5-di-*O*-monocaffeoylquinic through combining liquid chromatography and tandem mass spectrometry; and Weisz et al. [36] identified caffeic acid, ferulic acid, 5-*O*-feruloylquinic acid, and 5-*O*-*p*-coumaroylquinic acid via LC-MS. In this study, amino acids, such as tryptophan, arginine, isoleucine, valine, and tyrosine were also detected; these compounds are synthesised via the Shikimate pathway in plants [37]. Although linoelaidic acid and α-linolenic acid—two well-known fatty acids in sunflower seeds [7]—were identified in this study, they were present in low quantities. Based on the HPLC results, the presence of compounds in the sunflower seed extract was determined based on the retention time. Validation was performed by following the International Council for Harmonisation (ICH) guidelines based on our previous report [25]. This study found that chlorogenic acid was the most abundant phenolic acid in the sunflower seed extract (1.38 mg/g·DW), followed by caffeic acid (5.17 µg/g·DW). The chlorogenic acid content was in line with previous reports, varying from 500 µg/g·DW to 18.6 mg/g·DW [9,28,35,38]. The tryptophan and melatonin contents of 15.52 ± 0.04 µg/g·DW and 0.02 ± 0.00 µg/g·DW are consistent with reports stating that sunflower seeds are rich in tryptophan [39,40]. Interestingly, our results highlighted a melatonin content 40-fold higher than those found by Verde et al. [41] (0.5 ng/g) and Manchester et al. [42] (2–200 ng/g·DW). A *p*-coumaric acid peak was detected in the chromatogram, in a lower amount than the LOQ, while other compounds, including gallic acid, protocatechuic acid, *p*-hydroxybenzoic acid, syringic acid, ferulic acid, sinapic acid, rutin, quercetin, and serotonin, were not detected via LC-MS or HPLC. Phytochemical profiling revealed several compounds, including chlorogenic acid and tryptophan, that could promote wound healing. These compounds have been explored in previous studies. Bagdas et al. [14] found that chlorogenic acid may decrease nitric oxide levels, leading to accelerated wound healing in rats. Pugazhenthi et al. [43] reported that melatonin can promote angiogenesis and increase cytokine expression during tissue formation as part of the wound-healing processes. Tryptophan can accelerate wound healing in chronically stressed mice [44]. According to pharmacokinetic property prediction performed via ADMET, chlorogenic acid is predicted to show moderate absorption, better plasma distribution in plasma than tissue, with a log VDss 0.377; fast excretion, with total clearance of 0.378 log ml/min/kg, no carcinogenic effect, as determined via the Ames test; and no skin sensitisation (Table S4).

Combining various phytochemicals in the sunflower seed extract might lead to highly beneficial synergistic interactions that promote wound healing. Several studies have used network pharmacology to investigate the molecular mechanisms of wound healing [45,46,47]. Using this approach, we identified the top 10 hub targets (related to the inflammation, proliferation and remodelling phases) associated with the wound-healing activity of the sunflower seed extract. In the inflammation phase, cells secrete bioactive factors such as IL-6, CXCL8, and tumour necrosis factor (TNF); cells produce extracellular matrix components; and neutrophils and monocytes migrate to the lesion [48]. PTGS2 contributes to the regulation of the cellular responses to stimulus, cell differentiation, cell proliferation, and apoptosis. The NF-κB1, TNF, and MAPK signalling pathways promote the expression of cytokines, chemokines, and antimicrobial peptides [49]. Furthermore, NF-κB1 is found in almost all animal cells, and its overexpression could lead to chronic inflammation and delayed healing [45,50]. EGFR is vital for keratinocyte re-epithelialisation and regulated cellular processes such as cell proliferation and migration [1,51]. JUN regulates gene expression in response to stress and cytokines, and it is also involved in cell proliferation, differentiation, and apoptosis. Furthermore, AKT1 is part of the PI3K–AKT pathway, which promotes cell proliferation and survival [52]. These processes fill the wound. During the remodelling phase, MMP9 degrades extracellular matrix components, and inflammation and angiogenesis occur [53]. SRC regulates cell proliferation, differentiation, and survival and influences cell migration in response to growth factors [54]. Additionally, CASP3 helps to remove excess or damaged cells. These processes help to resolve inflammation and maintain cellular balance during tissue remodelling [46].

**Figure 6:**
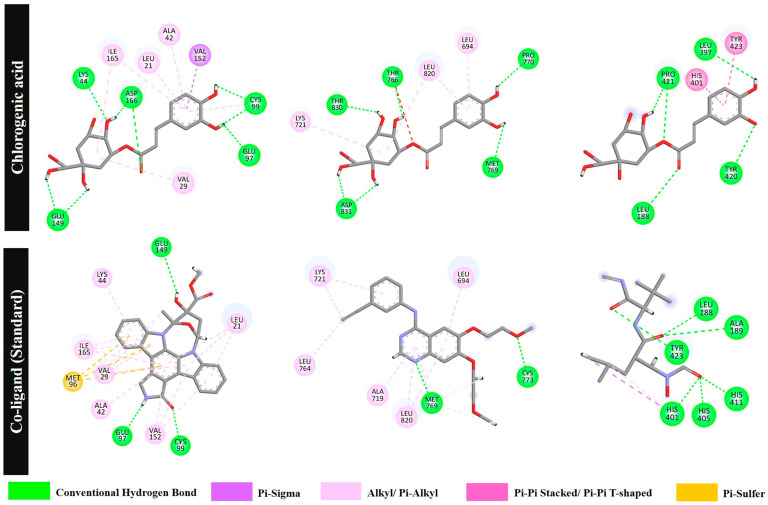
A two-dimensional visualisation of the molecular binding interactions of chlorogenic acid and the standard co-ligands with nuclear factor kappa B (NF-κB), the epidermal growth factor receptor (EGFR), and matrix metalloproteinase 9 (MMP9).

Based on molecular docking results (Figure 6), chlorogenic acid forms seven conventional hydrogen bonds with NF-κB (GLU97, GLU149, LYS44, ASP166, and CYS99) and exhibits a pi–sigma interaction with VAL152 and six pi–alkyl/alkyl interactions with VAL29, ILE165, LEU21, ALA42, and CYS99. The standard ligand KSA forms three strong hydrogen bonds with GLU97, GLU149, and CYS99; a pi–sulphur interaction with MET96; and six pi–alkyl/alkyl interactions with VAL29, ILE165, LEU21, ALA42, VAL152, and LYS44. Chlorogenic acid forms hydrogen bonds with GLU97 (1.82 Å) and CYS99 (2.06, 2.48 Å) located in the active site of NF-κB. Note that the ligand KSA also forms hydrogen bonds with GLU97 (1.72 Å) and CYS99 (1.77 Å) [55]. For EGFR, chlorogenic acid forms five conventional hydrogen bonds at MET769 (2.36 Å), PRO770 (2.86 Å), THR766 (2.05 Å), ASP831 (1.76, 1.73 Å), and THR830 (2.19 Å); one pi–sigma interaction with LEU694; and two pi–alkyl/alkyl interactions with LYS721 and LEU820. The standard ligand AQ4 forms two hydrogen bonds with MET769 (1.86 Å) and CYS773 (1.97 Å) (note the closer distance at MET769 compared with chlorogenic acid) and six pi–alkyl/alkyl interactions with LEU764, LYS721, ALA719, MET769, LEU820, and LEU694. Finally, for MMP9, chlorogenic acid forms hydrogen bonds with LEU188 (2.10 Å) and PRO421 (2.84 and 2.00 Å) in the binding pocket [47] and two pi–pi interactions with TYR423 and HIS401. The standard ligand NFH forms six conventional hydrogen bonds with LEU188, ALA189, HIS401, HIS405, HIS411, and TYR423; a pi-sigma interaction with HIS401; and one pi–alkyl interaction with HIS401. These findings suggest that chlorogenic acid, a major compound in the sunflower seed extract, might contribute to wound healing during multiple phases by interacting with multiple target proteins.

## 4. Materials and Methods

### 4.1. Preparation of the Sunflower Seed Extract

Sunflower seeds were purchased from a local market in Khon Kaen province, Thailand. The dried sample was macerated with methanol (AR grade, Fisher Scientific, Loughborough, UK) at a ratio of 1:2 (*w*/*v*) for 30 min using an ultrasonic bath (at a frequency of 28 KHz and 220 V) [56]. The methanol extract was subjected to vacuum filtration, and the residual was re-extracted twice following the same method. The filtrates were pooled, and methanol was evaporated using a rotary evaporator (Rotavapor^®^ Buchi model R-100, Flawil, Switzerland). The methanolic sunflower seed extract was stored at −20 °C until analysis.

### 4.2. Antioxidant Activity

#### 4.2.1. DPPH Assay

In brief, 100 µL of the extract and 100 µL of 200 µM DPPH (Fluka Chemikal, Buchs, Switzerland) were mixed in a 96-well plate. After incubation for 30 min in the dark, the absorbance at 517 nm was measured using a microplate reader (Sunrise Tecan, Grödig, Austria) [57]. Trolox (6-hydroxy-3,5,7,8-tetramethylchromane-2-carboxylic acid; Sigma-Aldrich, St. Louis, MO, USA) was used as a positive control. DPPH inhibition was calculated using Equation (1):

(1) $$\mathrm{Inhibition}\;\left(\%\right)\;=\frac{\left(\mathrm{Absorbance}\;\mathrm{of}\;\mathrm{control}\;-\;\mathrm{Absorbance}\;\mathrm{of}\;\mathrm{sample}\right)}{\mathrm{Absorbance}\;\mathrm{of}\;\mathrm{control}}\times \;100$$

IC_50_ was determined from a linear curve (plotting the percentage DPPH inhibition versus the concentration of the extract).

#### 4.2.2. ABTS Assay

The ABTS solution was prepared by combining 7 mM of ABTS (Sigma-Aldrich) and 2.45 mM of potassium persulphate (Sigma-Aldrich) and incubating the solution in the dark for 14–16 h. Then, the solution was diluted with distilled water (1:15, *v*/*v*). In a 96-well plate, the extract (100 µL) was mixed with freshly prepared ABTS solution (100 µL) and incubated in the dark for 30 min. The absorbance at 734 nm was read using a microplate reader [58]. Trolox was used as a positive control. The percentage of ABTS activity and the IC_50_ value were determined in the manner described in Section 4.2.1.

#### 4.2.3. FRAP Assay

The FRAP assay evaluated the ability of the sunflower seed extract to reduce Fe^3+^ (no colour) to Fe^2+^ (blue colour). The FRAP reagent was freshly prepared by mixing acetate buffer (pH 3.6), 10 mM of 2,4,6-Tris(2-pyridyl)-*s*-triazine (TPTZ, Fluka Chemikal, Buchs, Switzerland), and 20 mM of ferric chloride (Ajax Finechem, Taren Point, Australia) at a ratio of 10:1:1 (*v*/*v*/*v*). Then, 20 µL of extract was mixed with 80 µL of the FRAP reagent and incubated at room temperature in the dark. After 5 min, absorbance at 595 nm was determined [59]. Ferrous sulphate was used as a standard at a concentration range of 0–200 µM to generate a standard linear curve. FRAP is presented as mmole Fe^2+^/g·DW.

### 4.3. Evaluation of Wound Healing

#### 4.3.1. Cell Culture

HaCaT cells were purchased from the American Type Culture Collection (Manassas, VA, USA). They were maintained in Dulbecco’s Modified Eagle’s Medium (DMEM; Gibco Life Technologies, Grand Island, NY, USA) with 10% FBS (Gibco) and penicillin-streptomycin (10,000 U/mL; Gibco) at 37 °C and 5% CO_2_.

#### 4.3.2. Cell Viability Assay

HaCaT cells were seeded into a 96-well plate at an initial concentration of 1.5 × 10^5^ cells/mL in DMEM and incubated at 37 °C and 5% CO_2_ for 24 h. The adhered cells were treated with various concentrations of sunflower seed extract (10, 50, 100, and 200 µg/mL) for 24 h at 37 °C and 5% CO_2_. After removing the medium, MTT (Thermo Scientific, Waltham, MA, USA) was added, and the cells were incubated for 3 h at 37 °C and 5% CO_2_. Then, the medium was removed, and the formazan crystals were dissolved with dimethyl sulphoxide (DMSO, Thermo Scientific). The control was a medium derived from non-treated cells. The absorbance at 570 nm was read using a microplate reader (Ensight™, Perkin Elmer Inc., Waltham, MA, USA) [60]. Cell viability was calculated using Equation (2):

(2) $$\mathrm{Cell}\;\mathrm{viability}\;\left(\%\right)\;=\frac{\left({\mathrm{Absorbance}}_{\mathrm{Control}}-{\;\mathrm{Absorbance}}_{\mathrm{Sample}}\right)}{{\mathrm{Absorbance}}_{\mathrm{Control}}}\times \;100$$

#### 4.3.3. Wound Scratch Assay

The wound scratch assay was performed according to a method described by Loggenberg et al. [61], with slight modifications. HaCaT cells were seeded into a 24-well plate at a density of 1 × 10^5^ cells/mL (500 µL/well), incubated at 37 °C for 24 h, and cultured to a confluent monolayer. Scratches were created with a sterile 0.2 mL micropipette tip. The cells were washed with phosphate-buffered saline (PBS) to remove debris and treated with the sunflower seed extract (1 or 10 µg/mL). The cells were imaged after 24, 48, and 72 h using the EVOS™ FL Auto 2 Imaging System (4× magnification; Thermo Scientific). The percentage wound closure was analysed using ImageJ software (version 1.53e) by measuring the wound area at the beginning of treatment (0 h) and then every 24 h until closure. The percentage of wound closure was calculated using Equation (3):

(3) $$\mathrm{Wound}\;\mathrm{closure}\;\left(\%\right)\;=\frac{\left(\mathrm{Initial}\;\mathrm{wound}\;\mathrm{area}\;-\;\mathrm{Wound}\;\mathrm{area}\;\mathrm{at}\;\mathrm{each}\;\mathrm{time}\;\mathrm{point}\right)}{\mathrm{Initial}\;\mathrm{wound}\;\mathrm{area}}\times \;100$$

### 4.4. Phytochemical Profiling Analysis

#### 4.4.1. LC-MS

Phytochemical profiling of the sunflower seed extract was performed using a 1290 Infinity II LC instrument coupled to an LC-QTOF 6545XT instrument (both from Agilent Technologies, Santa Clara, CA, USA). The sunflower seed extract was diluted with 70% methanol containing 50 ng/mL of sulphadimethoxine as an internal standard to a final concentration of 10 mg/mL. The compounds in the sunflower seed extract were subjected to gradient elution using a Poroshell 120 EC-C18 column (2.1 × 100 mm, Agilent Technologies, Santa Clara, CA, USA) at 50 °C. The injection volume was 10 µL and the flow rate was 0.4 mL/min. The solvent consisted of 0.1% formic acid in water (mobile phase A) and 0.1% formic acid in acetonitrile (mobile phase B). The gradient elution was as follows: 0–0.5 min, 100% mobile phase A; 0.5–10.5 min, 0–55% mobile phase B; 10.5–12.5 min, 55–75% mobile phase B; 12.5–14 min, 75–100% mobile phase B; 14–17 min; 100% mobile phase B; 17–17.5 min, 100–0% mobile phase B; and 100% mobile phase A for re-equilibration. Mass spectrometry with high-resolution detection was run in positive and negative modes. The electrospray ionisation (ESI) conditions were set using drying gas at 325 °C at a flow rate of 13 L/h, and sheath gas at 275 °C at a flow rate of 12 L/h. The nebuliser pressure was 45 psi. The capillary voltages for the positive and negative modes were 4000 and 3000 V, respectively. The instrument was operated in the MS1 and MS2 mass ranges at 40–1700 *m*/*z* and 25–1000 *m*/*z*, respectively. The collision energy was set at 20 eV and at 10 eV for the positive mode, with an acquisition rate of 3.35 spectra/s.

The raw LC-MS data were analysed using MS-Dial software (version 5.3), with normalisation to the internal standard (sulphadimethoxine). The data were pre-filtered using the following criteria: features with a retention time between 0.2 and 18 min, a mass error ≤ ±20 ppm, and an identification score ≥0.70 were retained [23,24]. To minimise background noise, mass exclusion was set at 112.9856, 119.0363, 966.0007, and 1033.9881 *m*/*z* in the negative mode and at 121.0509 and 922.0098 *m*/*z* in the positive mode. The focus was on identifying phenolic acids, flavonoids, and fatty acids. The spectra were compared to various reference libraries, including MS-Dial ESI(+/−) MS/MS from authentic standards, the Fiehn/Vaniya natural product, and the Bioinformatic & Molecular Design Research Center Mass Spectral Library—Natural Products.

#### 4.4.2. Determination of the TPC and the TFC

The TPC was measured using the Folin–Ciocalteu colorimetric method. In a 96-well plate, 20 μL of extract was mixed with 10% Folin–Ciocalteu reagent (LOBA Chemis, Mumbai, India), and incubated in the dark for 5 min. Then, 80 μL of 7% sodium carbonate (Ajax Finechem) was added [62]. After incubation for 30 min, the absorbance at 700 nm was read using a microplate reader. The TPC was calculated from the standard gallic acid linear curve and is presented as mg GAE/g·DW.

The TFC was measured by mixing the extract (100 µL) and 2% aluminium chloride (100 µL) in a 96-well plate. After incubation for 30 min, the absorbance at 415 nm was measured [62]. Quercetin was used as a standard to generate a standard curve. The TFC is measured in mg QUE/g·DW.

#### 4.4.3. HPLC

Phenolic compounds were analysed via HPLC using a Prominence-I LC-2030C 3D apparatus (Shimadzu Co., Kyoto, Japan) with a diode array detector. The samples were separated using a C18 column (250 mm × 4.6 mm, 5 µm, Unisol model, Bonna-Agela Technologies, Wilmington, DE, USA) with a gradient system at a flow rate of 0.8 mL/min. Mobile phase A was 1% acetic acid (in deionised water), and mobile phase B was acetonitrile. The extracts were analysed at a wavelength of 280 nm for gallic acid, protocatechuic acid, *p*-hydroxybenzoic acid, and syringic acid; at 320 nm for chlorogenic acid, caffeic acid, *p*-coumaric acid, ferulic acid, and sinapic acid; and at 370 nm for rutin and quercetin. The limit of detection and limit of quantification of the phenolic compounds were 0.001 and 0.01 μg/mL, respectively [25]. The analysis was repeated three times, and the results are reported as mg/g·DW.

The indolamine compounds, including tryptophan and melatonin, were examined via HPLC with a fluorescence detector. The extract was concentrated via solid phase extraction using VertiPak™ C18-SPE (Vertical Chromatography Co., Nonthaburi, Thailand) and further dried under nitrogen gas. The sample and standards were separated using a Unisol C18 column (250 mm × 4.6 mm, 5 µm, Bonna-Agela Technologies) at a flow rate of 1.0 mL/min. Detection involved an excitation wavelength of 286 nm and an emission wavelength of 346 nm. The limit of detection was 0.0025 μg/mL and the limit of quantification was 0.05 μg/mL [25]. The data are presented as μg/g·DW.

### 4.5. Network Pharmacology

#### 4.5.1. Screening of Gene Targets of Compounds in the Sunflower Seed Extract

The 47 compounds found in the sunflower seed extract via LC-MS and HPLC analysis were used for target prediction via SwissTargetPrediction (http://www.swisstargetprediction.ch/, accessed on 13 June 2025) and Similarity Ensemble Approach (https://sea.bkslab.org/, accessed on 14 June 2025). The species was set as *Homo sapiens* and the predicted probability value was >0.1 of the collected targets. For Similarity Ensemble Approach, the target gene IDs were matched to the UniProt IDs via the ID mapping tool in the UniProt database (https://www.uniprot.org/, accessed on 13 June 2025). The collected targets were merged, and duplicates were removed. Finally, the common targets between SwissTargetPrediction and Similarity Ensemble Approach were identified using a Venn diagram, which was prepared using Venny 2.1 (https://bioinfogp.cnb.csic.es/tools/venny/index.html, accessed on 14 June 2025). To identify therapeutic targets, GeneCards (https://www.genecards.org/, accessed on 14 June 2025) was searched using the keyword “wound healing”. A Venn diagram was prepared using Venny 2.1 to determine the overlap between the sunflower seed extract targets and the therapeutic targets.

#### 4.5.2. PPI Network Construction

The STRING database (https://cn.string-db.org/, version 12.0, accessed on 23 June 2025) was used for network analysis [63]. The parameters were as follows: *H. sapiens* was set as the organism, evidence for network edges was restricted to high-confidence interactions (score > 0.7), the FDR stringency was set to medium, and disconnected nodes were excluded from the network. The STRING data were imported to Cytoscape (version 3.10.3) to visualise the PPI network. Furthermore, the CytoHubba plugin was used to screen the top 10 hub target proteins based on degree, betweenness, and closeness.

#### 4.5.3. GO and KEGG Pathway Analyses

The STRING database (accessed on 23 June 2025) was used for GO and KEGG pathway enrichment analysis. The parameters were as follows: *H. sapiens* was set as the organism and the FDR cutoff was 0.01. The KEGG pathways related to wound healing were used to generate a Sankey diagram using SRplot software [64].

### 4.6. Molecular Docking Study

The molecular docking analysis was run using MGLTools software (version 1.5.7) from Auto-Dock 4.2 [65]. The X-ray crystal structures of NF-κB (PDB ID 4KIK), EGFR (PDB ID 1M17), and MMP9 (PDB IDs: and 1GKC) were retrieved from the RCSB protein data bank (https://www.rcsb.org/, accessed on 13 June 2025) [55,66,67]. First, the proteins were prepared in a protonated state on the PDB2PQR website [68]. All polar hydrogen atoms were added to the proteins, and Kollman charges were designated to signify electrostatic forces between the protein and the ligand. The chemical structure of the ligands was prepared using Marvin. The ligand was automatically maximised via AutoDock Tools (v.1.5.7) and saved as a pdbqt file. The crystal structure coordinates were converted to the pdbqt format for docking analysis. The Lamarckian genetic algorithm was used for all computations. The validation process included redocking the natural ligand back onto its corresponding receptor. Docking precision was evaluated based on the root-mean-square deviation, with a value of <2.0 Å indicative of good docking (Table 6) [69]. The structure exhibiting the lowest binding energy and a suitable conformation was selected for further evaluation. The accuracy of the docking positions was verified by visual observation using BIOVIA Discovery Studio Client 2020.

### 4.7. Pharmacokinetic Predictions

The pkCSM online platform (https://biosig.lab.uq.edu.au/pkcsm/prediction; accessed on 30 October 2025) [26] was used to predict the absorption (A), distribution (D), metabolism (M), excretion (E), and toxicity (T) properties of the compounds involved in the molecular docking analysis.

### 4.8. Statistical Analysis

The experiments were carried out in triplicate, and the data are presented as the mean ± standard deviation. The data were analysed via one-way analysis of variance, followed by Tukey’ test for pairwise comparisons, with *p* < 0.05 considered to indicate a statistically significant difference.

## 5. Conclusions

This study found that sunflower seed extract exhibited moderate-to-strong antioxidant activity, revealing the wound-healing activity of this extract using a HaCaT cell model. A concentration of 10 μg/mL promoted HaCaT cell migration, leading to full wound closure within 24 h after injury. According to phytochemical profiling via LC-MS and HPLC analyses, chlorogenic acid was found to be a major compound in the extract, along with caffeic acid, *p*-coumaric acid, tryptophan, and melatonin. The identified compounds were chosen for analysis through network pharmacology and molecular docking to understand the mechanisms through which the sunflower seed extract promotes wound healing. The top 10 hub targets were NF-κB1, EGFR, MMP9, JUN, SRC, IL6, AKT1, PTGS2, CXCL8, and CASP3. Among these, NF-κB, EGFR, and MMP9 were selected for molecular docking; these targets represent the inflammation, proliferation, and remodelling phases of wound healing, respectively. Our findings revealed chlorogenic acid to be a major component of sunflower seed extract, which may be responsible for its wound-healing activity. Chlorogenic acid interacts strongly with NFκB1, EGFR, and, especially, MMP9 (with stronger binding affinity than the standard ligand). Furthermore, its pharmacokinetic properties are predicted to show moderate absorption, good distribution, and no predicted carcinogenic or skin-sensitising effects. Taking these results together highlights the potential of the sunflower seed extract to be developed as a therapeutic option for wound healing.

## Figures and Tables

**Table 1:** The identification of compounds in the sunflower seed extract in the negative electrospray ionisation mode (analysed using MS-Dial software).

Retention Time (min)	Compound	Adduct	Theoretical (*m*/*z*)	Observed (*m*/*z*)	Mass Error (ppm)	Intensity (×10^5^)	Relative (%)
0.657	D-Gluconic acid	[M − H]^−^	196.16	195.05	−2.31	0.73	0.11
1.238	Pyroglutamic acid	[M − H]^−^	129.11	128.03	0.08	9.32	1.37
2.646	Xanthine	[M − H]^−^	152.11	151.03	−6.89	9.09	1.34
2.656	Xanthosine	[M − H]^−^	284.23	283.07	−0.64	16.7	2.46
2.764	L-(-)-Phenylalanine	[M − H]^−^	165.19	164.07	−1.1	0.90	0.13
3.250	Chlorogenic acid	[M − H]^−^	354.31	353.09	1.47	157	23.21
3.392	Tryptophan	[M − H]^−^	204.22	203.08	2.27	2.30	0.34
3.667	Bergenin	[M − H]^−^	328.27	327.07	−3.00	0.11	0.02
3.776	2-Isopropylmalic acid	[M − H]^−^	176.17	175.06	−1.31	3.11	0.46
3.839	D-(-)-Quinic acid	[M − H]^−^	192.17	191.06	−8.43	319	47.03
3.941	Glutamylphenylalanine	[M − H]^−^	294.30	293.12	−0.41	2.86	0.42
4.024	Caffeic acid	[M − H]^−^	180.16	179.03	−1.06	17.7	2.60
4.597	Agnuside	[M − H]^−^	466.40	465.14	−6.11	4.01	0.59
4.732	Tricin 5-glucoside	[M − H]^−^	492.40	491.12	2.69	22.5	3.32
5.056	Umbelliferone	[M − H]^−^	162.14	161.02	0.93	2.93	0.43
5.368	Plantaginin	[M − H]^−^	448.40	447.09	0.34	0.34	0.05
5.584	4-Hydroxyquinoline	[M − H]^−^	145.16	144.05	0.42	1.28	0.19
5.634	Phenylacetic acid	[M − H]^−^	136.15	135.05	−8.74	2.80	0.38
5.907	3,4-Di-*O*-caffeoylquinic acid	[M − H]^−^	516.40	515.12	−7.11	17.3	9.89
5.966	Eriodictyol	[M − H]^−^	288.25	287.06	0.63	1.66	0.25
5.976	Isookanin-7-*O*-glucoside	[M − H]^−^	450.40	449.11	1.22	17.9	2.64
6.007	Azelaic acid	[M − H]^−^	188.22	187.10	1.98	3.13	0.46
12.632	α-Linolenic acid	[M − H]^−^	278.40	277.22	4.73	4.53	0.67
14.214	16-Hydroxyhexadecanoic acid	[M − H]^−^	272.42	271.23	−16.41	2.27	0.33
14.567	Linoelaidic acid	[M − H]^−^	280.40	279.23	2.08	8.91	1.31

**Table 2:** The identification of compounds in the sunflower seed extract in the positive electrospray ionisation mode (analysed with the MS-Dial software).

Retention Time (min)	Compound	Adduct	Theoretical (*m*/*z*)	Observed (*m*/*z*)	Mass Error (ppm)	Intensity (×10^5^)	Relative (%)
0.702	Trigonelline	[M + H]^+^	137.14	138.06	−15.94	38.3	5.13
0.737	Arginine	[M + H]^+^	174.20	175.12	−1.83	1.14	0.15
0.780	Isoleucine	[M + H]^+^	131.17	132.10	−6.59	4.56	0.61
0.934	Valine	[M + H]^+^	117.15	118.09	−3.13	11.1	1.48
0.982	4-Guanidinobutyric acid	[M + H]^+^	145.10	146.09	4.04	1.17	0.16
1.514	Tyrosine	[M + H]^+^	181.19	182.08	2.58	3.65	0.49
2.654	4-Methyl-5-thiazoleethanol	[M + H]^+^	143.21	144.05	−2.22	34.9	4.67
2.778	7,8-Dimethoxycoumarin	[M + H]^+^	206.19	207.07	−3.82	285	38.09
2.998	1,2,3,9-Tetrahydro-4H- carbazol-4-one	[M + Na]^+^	185.22	208.07	4.95	21.0	2.81
3.409	*p*-Coumaric acid	[M + H]^+^	164.16	165.05	3.76	3.49	0.47
3.422	2-Naphthylamine	[M + H]^+^	143.18	144.08	−10.9	19.4	2.59
3.864	Chlorogenic acid	[M + Na]^+^	354.31	377.09	0.74	145	19.37
3.936	Caffeoylcholine	[M]^+^	266.31	266.14	−9.17	85.4	11.43
3.974	4-Coumaroylcholine	[M]^+^	250.31	250.14	−6.96	18.9	2.53
5.250	(+)Catechin	[M + H]^+^	290.27	291.09	12.06	1.02	0.14
5.307	1-Ethyl-9H-pyrido [3,4-b] indole	[M + H]^+^	196.25	197.11	−0.71	3.52	0.47
5.503	*N*-Acetyltryptophan	[M + H]^+^	246.26	247.11	1.17	2.61	0.35
5.925	3,4-Di-*O*-caffeoylquinic acid	[M + Na]^+^	516.40	539.12	5.79	10.5	1.40
5.990	Eriodictyol	[M + H]^+^	288.25	289.07	−4.22	9.58	1.28
6.159	Cimifugin 4′-*O*-beta-D- glucopyranoside	[M + Na]^+^	468.40	491.15	−7.78	17.0	2.28
6.453	Skimmin	[M + H]^+^	324.28	325.09	−0.74	10.9	1.45
8.426	Pinoresinol 4-*O*-glucoside	[M + Na]^+^	520.50	543.18	−6.74	2.33	0.31
12.454	Phosphocholine	[M + H]^+^	183.14	184.07	2.01	17.6	2.35

**Table 3:** The quantification of select phenolic acids and indolamines in the sunflower seed extract based on high-performance liquid chromatography.

Phytochemical	Retention Time (min)	Standard Equation (R^2^)	Content
**Phenolic acids**			
Chlorogenic acid	19.739	y = 3428.8x − 591.22 (R^2^ = 0.9999)	1.38 ± 0.02 mg/g·DW
Caffeic acid	23.633	y = 9810.5x − 3571.8 (R^2^ = 0.9995)	5.17 ± 0.06 µg/g·DW
*p*–Coumaric acid	30.890	y = 7749.8x − 4117.6 (R^2^ = 0.9994)	NQ
**Indolamines**			
Tryptophan	8.338	y = 1.8051x + 0.0064 (R^2^ = 0.9993)	15.52 ± 0.04 µg/g·DW
Melatonin	30.793	y = 6.5972x + 0.1177 (R^2^ = 0.9973)	0.02 ± 0.00 µg/g·DW

The data are expressed as mean ± standard deviation (n = 3). DW = dry weight. NQ = not quantified (the value was <0.21 µg/g·DW).

**Table 4:** The details of the enriched pathways related to wound healing (based on Kyoto Encyclopaedia of Genes and Genomes [KEGG] enrichment analysis).

KEGG ID	Signalling Pathway	False Discovery Rate *p*-Value	Target Genes
hsa04510	Focal adhesion	1.15 × 10^−9^	CCND1, ITGA2B, PRKCG, BIRC3, EGFR, MET, ROCK2, GSK3B, XIAP, JUN, SRC, GRB2, ITGB1, ITGA4, ROCK1, HRAS, PRKCA, AKT1, ITGB3
hsa04657	IL-17 signalling pathway	9.82 × 10^−8^	NFκB1, MMP13, MMP3, CXCL8, CASP3, MMP1, GSK3B, CASP8, PTGS2, JUN, MMP9, IL6
hsa01521	EGFR tyrosine kinase inhibitor resistance	1.84 × 10^−7^	PRKCG, FGF2, EGFR, MET, GSK3B, SRC, GRB2, IL6, HRAS, PRKCA, AKT1
hsa00590	Arachidonic acid metabolism	2.11 × 10^−7^	LTA4H, ALOX12, CBR1, PTGS1, PLA2G4A, PTGS2, CYP2C19, ALOX5, AKR1C3, PLA2G2A
hsa04015	Rap1 signalling pathway	2.11 × 10^−7^	ITGA2B, PRKCG, FGF2, EGFR, MET, DRD2, CNR1, LPAR3, SRC, ITGB1, HRAS, PRKCA, AKT1, ITGB3, FPR1, FGF1
hsa04668	TNF signalling pathway	5.18 × 10^−7^	NFκB1, BIRC3, MMP3, CASP3, SELE, CASP8, PTGS2, CASP7, JUN, MMP9, IL6, AKT1
hsa04370	VEGF signalling pathway	1.05 × 10^−6^	PRKCG, NOS3, MAPKAPK2, PLA2G4A, PTGS2, SRC, HRAS, PRKCA, AKT1
hsa04611	Platelet activation	1.08 × 10^−6^	ITGA2B, NOS3, F2, ROCK2, PTGS1, PLA2G4A, SRC, ITGB1, ROCK1, TBXA2R, AKT1, ITGB3
hsa04218	Cellular senescence	1.20 × 10^−6^	SIRT1, NFKB1, CCND1, CDC25A, CXCL8, MAPKAPK2, IGFBP3, HLA-A, IL6, HRAS, CAPN1, AKT1, CCNA2
hsa04151	PI3K–Akt signalling pathway	1.77 × 10^−6^	NFKB1, IL2, CCND1, ITGA2B, FGF2, EGFR, NOS3, MET, GSK3B, LPAR3, GRB2, ITGB1, ITGA4, IL6, HRAS, PRKCA, AKT1, ITGB3, FGF1
hsa04010	MAPK signalling pathway	2.34 × 10^−6^	NFκB1, PRKCG, FGF2, EGFR, CASP3, MET, MAPT, MAPKAPK2, PLA2G4A, JUN, HSPA1B, RPS6KA3, GRB2, HRAS, PRKCA, AKT1, FGF1
hsa04066	HIF-1 signalling pathway	9.33 × 10^−6^	HMOX1, NFκB1, PRKCG, EGFR, NOS3, NOS2, EGLN1, IL6, PRKCA, AKT1
hsa04012	ErbB signalling pathway	1.17 × 10^−5^	PRKCG, EGFR, GSK3B, JUN, SRC, GRB2, HRAS, PRKCA, AKT1
hsa04540	Gap junction	1.92 × 10^−5^	HTR2B, PRKCG, EGFR, DRD2, SRC, GRB2, HRAS, PRKCA, HTR2A
hsa04014	Ras signalling pathway	4.8 × 10^−5^	NFκB1, PRKCG, FGF2, EGFR, MET, PLA2G4A, GRB2, PLA2G2A, HRAS, PRKCA, AKT1, FGF1, PTPN11
hsa04062	Chemokine signalling pathway	1.60 × 10^−4^	GSK3A, NFKB1, CXCL8, ROCK2, GSK3B, PRKCD, SRC, GRB2, ROCK1, HRAS, AKT1
hsa04310	Wnt signalling pathway	1.70 × 10^−4^	CCND1, MMP7, PRKCG, PPARD, CTBP2, ROCK2, GSK3B, PSEN1, JUN, PRKCA
hsa04630	JAK–STAT signalling pathway	2.00 × 10^−4^	IL2, CCND1, EGFR, PTPN2, GRB2, IL6, PTPN6, HRAS, AKT1, PTPN11
hsa04064	NF-kappa B signalling pathway	5.50 × 10^−3^	NFκB1, BIRC3, CXCL8, PARP1, PTGS2, XIAP

**Table 5:** The binding free energy (ΔG) of the main compounds in the sunflower seed extract and co-ligands with the hub targets NF-κB, EGFR, and MMP9.

Compound	Compound Name	ΔG (kcal/mol)		
		NF-κB	EGFR	MMP9
**1**	Pyroglutamic acid	−4.34	−3.63	−3.89
**2**	Xanthine	−4.56	−3.92	−5.17
**3**	Xanthosine	−5.98	−5.84	−6.34
**4**	D-(-)-quinic acid	−3.77	−3.84	−3.81
**5**	Caffeic acid	−5.34	−4.97	−5.24
**6**	Tricin 5-glucoside	−10.32	−6.93	−6.97
**7**	3,4-Di-*O*-caffeoylquinic acid	−9.16	−6.43	−7.08
**8**	Isookanin-7-*O*-glucoside	−10.06	−6.82	−8.65
**9**	Linoelaidic acid	−6.90	−5.60	−5.63
**10**	Trigonelline	−4.21	−4.03	−3.89
**11**	Valine	−4.04	−4.23	−4.65
**12**	4-Methyl-5-thiazoleethanol	−4.25	−4.33	−5.18
**13**	7,8-Dimethoxycoumarin	−5.92	−5.30	−6.49
**14**	1,2,3,9-Tetrahydro-4H-carbazol-4-one	−6.84	−5.75	−8.14
**15**	2-Naphthylamine	−5.45	−5.38	−6.35
**16**	Chlorogenic acid	−8.12	−6.73	−8.90
**17**	Caffeoylcholine	−6.37	−5.54	−7.85
**18**	4-Coumaroylcholine	−6.19	−5.62	−8.21
**19**	Eriodictyol	−8.71	−7.12	−8.29
**20**	Cimifugin 4′-*O*-beta-D-glucopyranoside	−7.43	−7.12	−9.41
**21**	Skimmin	−3.30	−2.76	−4.00
-	Co-ligands *	−11.90	−7.00	−7.87

* The co-ligands are KSA for NF-κB, AQ4 for EGFR, and NFH for MMP9.

**Table 6:** The re-docking of the co-ligand interactions with the three evaluated protein targets involved in the wound-healing process.

Protein	Co-Ligand	Grid Box Size (Å)	Spacing (Å)	Root-Mean-Square Deviation (Å)
NF-κB	KSA or K-252A	X = 48.383 Y = 30.057 Z = −56.809	0.375	0.951
EGFR	AQ4 or [6,7-Bis(2-methoxy-ethoxy)quinazoline-4-yl]- (3-ethynylphenyl)amine	X = 22.013 Y = 0.252 Z = 52.794	0.477	1.775
MMP9	NFH or N~2~-[(2R)-2-{[formyl(hydroxy)amino]methyl}-4-methylpentanoyl]-N,3-dimethyl-L-valinamide	X = 65.607 Y = 31.083 Z = 117.697	0.408	1.651

## Data Availability

The original contributions presented in this study are included in this article.

## Supplementary Materials

The following supporting information can be downloaded at https://www.mdpi.com/article/10.3390/plants15020187/s1. Supplementary data to this article can be found in Supplementary files as follows: Supplementary Material S1 contains the relevant figures and tables, Figure S1 (Antioxidant activities of sunflower seed (SS) extract (left) and standard Trolox (right) on (A) ABTS assay and (B) DPPH assay), Figure S2 (The whole LC-MS chromatograms in both negative and positive ionization modes of sunflower seed extract obtained from LC-QTOF-MS analysis), Figure S3 (Chromatogram of standard phenolic compounds at a concentration of 500 µg/mL and the methanolic sunflower seed extract analyzed by HPLC-DAD represented at 320 nm), Figure S4 (Chromatogram of standard serotonin, tryptophan, and melatonin at a concentration of 4 µg/mL and the methanolic sunflower seed extract analyzed by HPLC-FLD), Figure S5 (2D Visualization of molecular binding interactions of compounds with top 5 binding score and co-ligand (standard) against NF-κB target), Figure S6 (2D Visualization of molecular binding interactions of compounds with top 5 binding score and co-ligand (standard) against EGFR target), Figure S7 (2D Visualization of molecular binding interactions of compounds with top 5 binding score and co-ligand (standard) against MMP9 target), Table S1 (All compound identification in SS extract under negative electrospray ionization (ESI) mode analyzed by MS-Dial software), Table S2 (All compound identification in SS extract under positive electrospray ionization (ESI) mode analyzed by MS-Dial software), Table S3 Binding interactions of 21 compounds form the SS extract corresponding NF-κB, EGFR, and MMP9 proteins involved in a wound healing, Table S4 (The selected ADMET parameters for the compounds obtained from pkCSM online tool). Additionally, the raw LC-MS data are provided in Supplementary Material S2.
