# Supplementary material for: Network Pharmacology of the Phytochemical Content of Sunflower Seed (Helianthus annuus L.) Extract from LC-MS on Wound-Healing Activity and the In Vitro Wound Scratch Assay
Source: Plants (Basel). 2026 Jan 7;15(2):187. doi: 10.3390/plants15020187 (PMC12845310; doi:10.3390/plants15020187)
Supplement: Supplementary file 1 [file plants-15-00187-s001.zip › Supplementary Material S1-FIgures and Tables.pdf]

Network Pharmacology of the Phytochemical Content of Sunflower Seed (*Helianthus annuus* L.) Extract from LC-MS on Wound-Healing Activity and the In Vitro Wound Scratch Assay

Juthamat Ratha [1], Tanit Padumanonda [2], Chawalit Yongram [3], Pimolwan Siriparu [4], Suthida Datham [4], Muhammad Subhan [4], Chatchavarn Chenboonthai [5] and Ploenthip Puthongking [1,6,*]

[1] Melatonin Research Group, Khon Kaen University, Khon Kaen 40002, Thailand; juthra@kku.ac.th
[2] Division of Pharmacognosy and Toxicology, Faculty of Pharmaceutical Sciences, Khon Kaen University, Khon Kaen 40002, Thailand; tanpad@kku.ac.th
[3] Radiation Dose Assessment Section, Regulatory Technical Support Division, Office of Atoms for Peace, Bangkok 10900, Thailand; chawalit.y@oap.go.th
[4] Faculty of Pharmaceutical Sciences, Graduate School, Khon Kaen University, Khon Kaen 40002, Thailand; pimolwan.s@kkumail.com (P.S.); suthida.tham@kkumail.com (S.D.); muhammad.s@kkumail.com (M.S.)
[5] General Drugs House Co., Ltd., 90, 90/1 Moo 4, Bueng Kham Phroi District, Lam Luk Ka 12150, Thailand; chatchvarn@yahoo.com
[6] Division of Pharmaceutical Chemistry, Faculty of Pharmaceutical Sciences, Khon Kaen University, Khon Kaen 40002, Thailand
* Correspondence: pploenthip@kku.ac.th

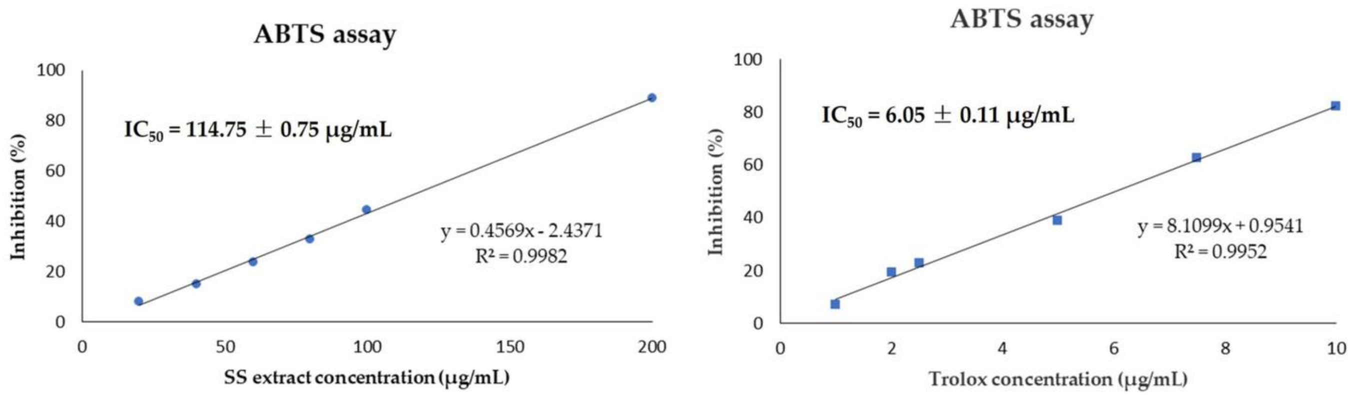

(A)

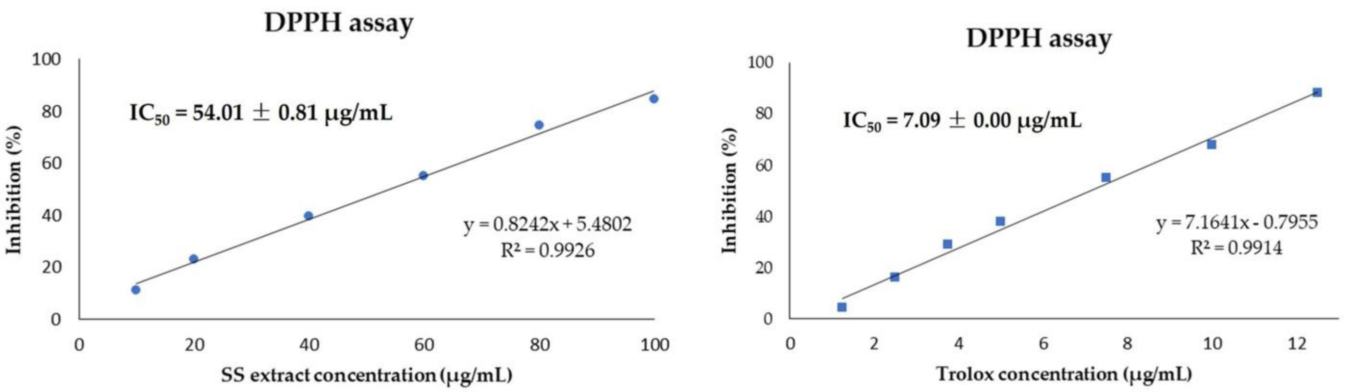

(B)

Figure S1. Antioxidant activities of sunflower seed (SS) extract (left) and standard Trolox (right) on (**A**) ABTS assay and (**B**) DPPH assay (n = 3).

Negative mode

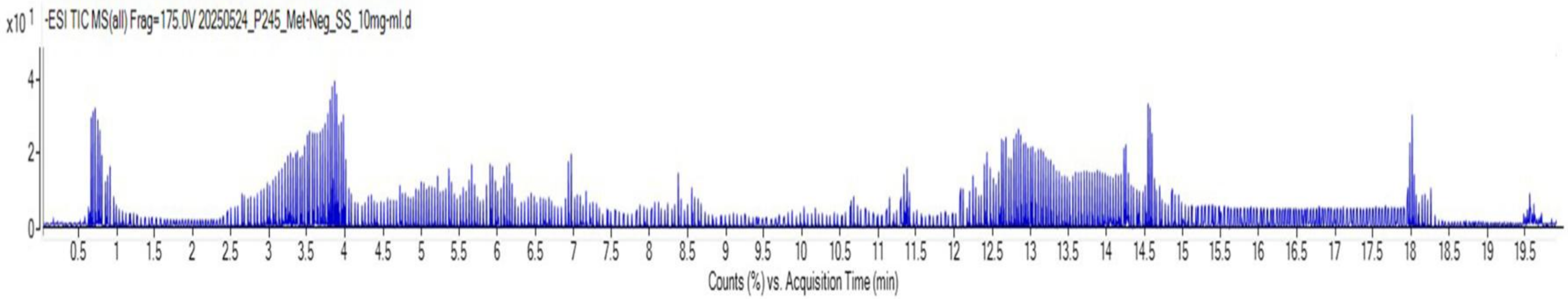

Positive mode

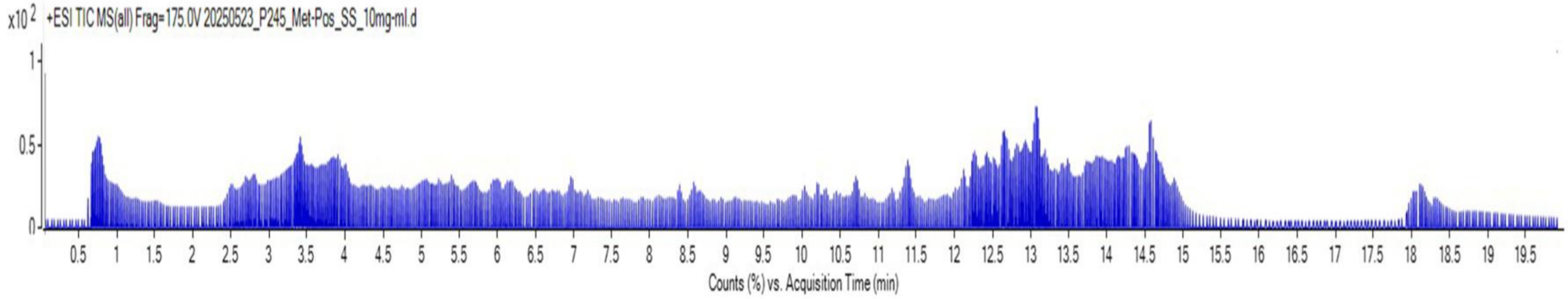

Figure S2. The whole LC-MS chromatograms in both negative and positive ionization modes of sunflower seed extract obtained from LC-QTOF-MS analysis.

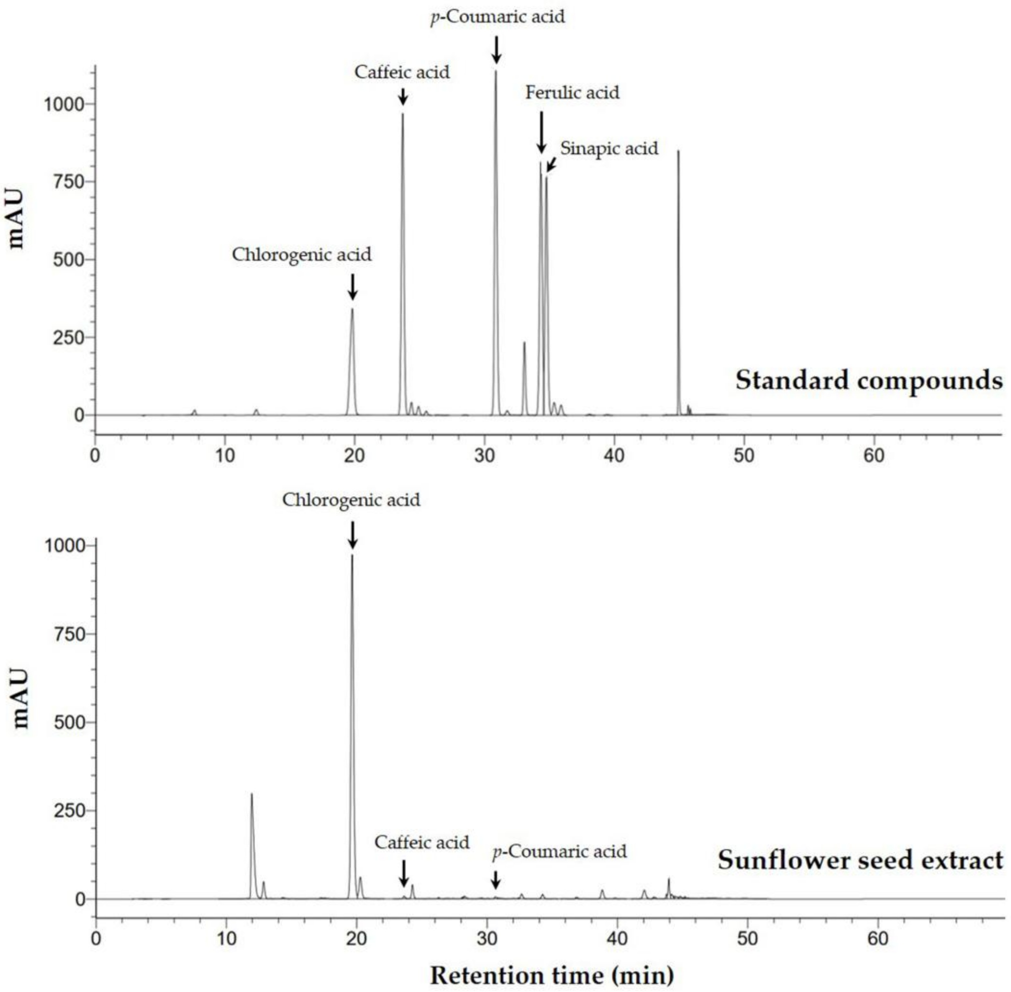

Figure S3. Chromatogram of standard phenolic compounds at a concentration of 500 μg/mL and the methanolic sunflower seed extract analyzed by HPLC-DAD represented at 320 nm (n = 3).

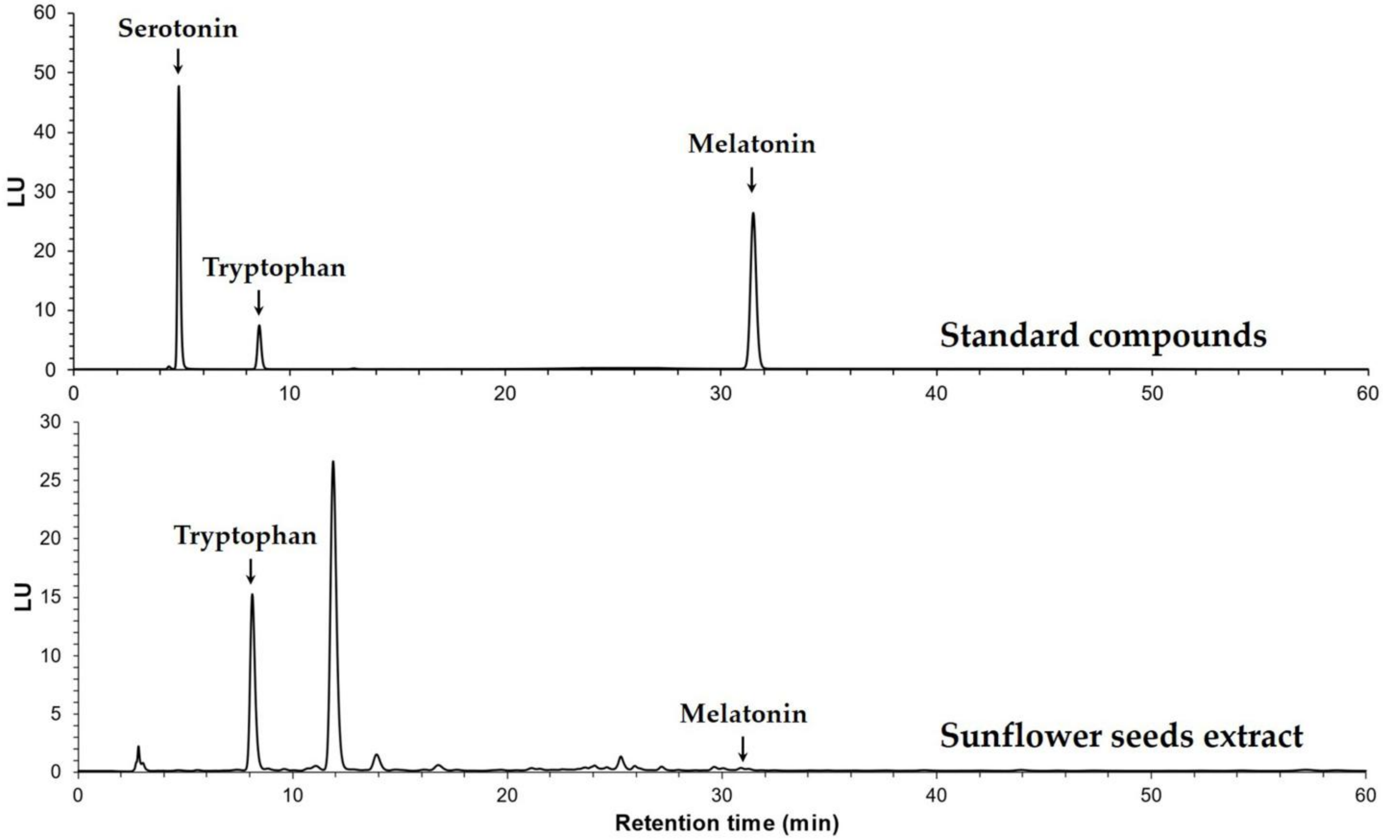

Figure S4. Chromatogram of standard serotonin, tryptophan, and melatonin at a concentration of 4 μg/mL and the methanolic sunflower seed extract analyzed by HPLC-FLD (n = 3).

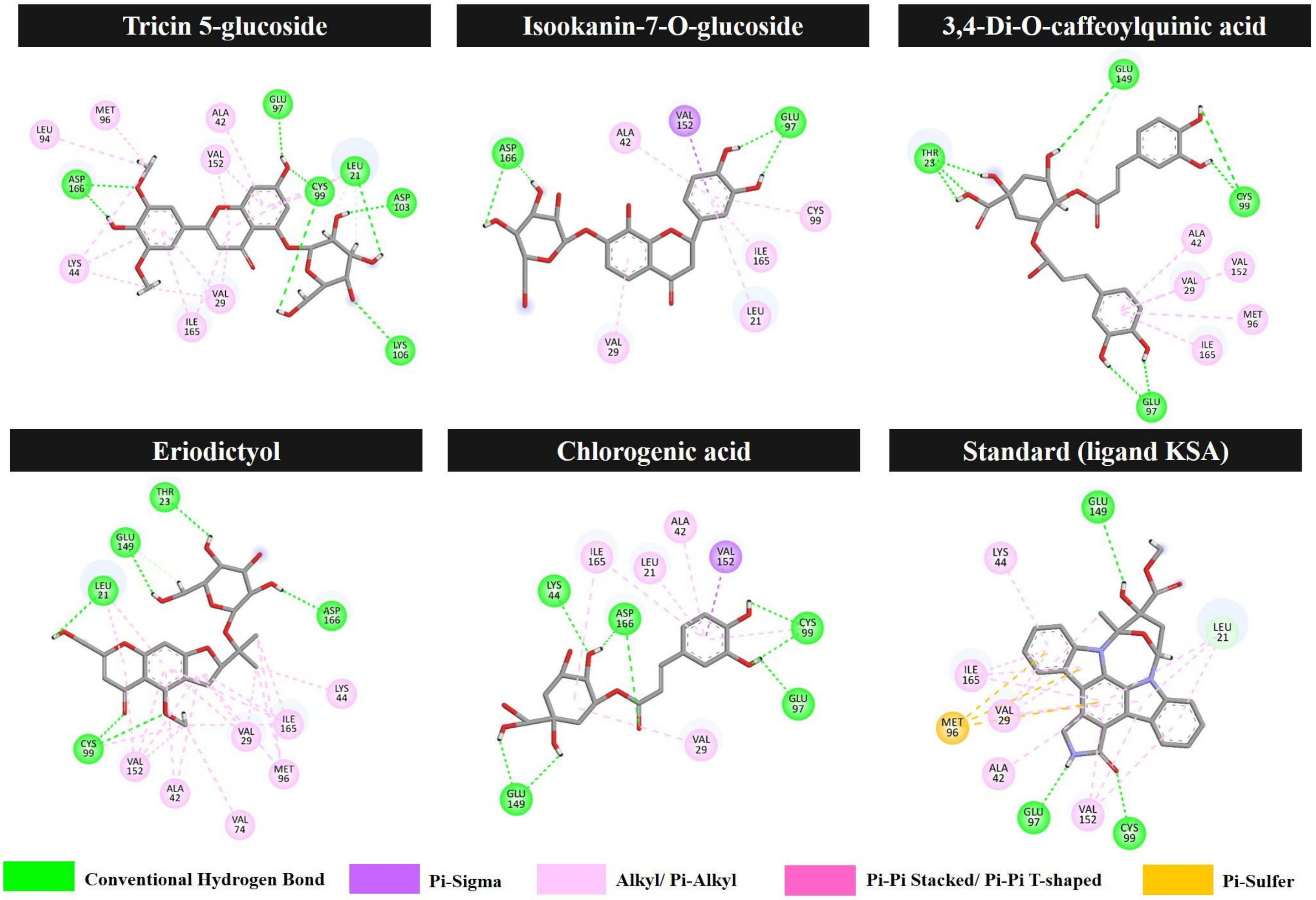

Figure S5. 2D Visualization of molecular binding interactions of compounds with top 5 binding score and co-ligand (standard) against NF-κB target.

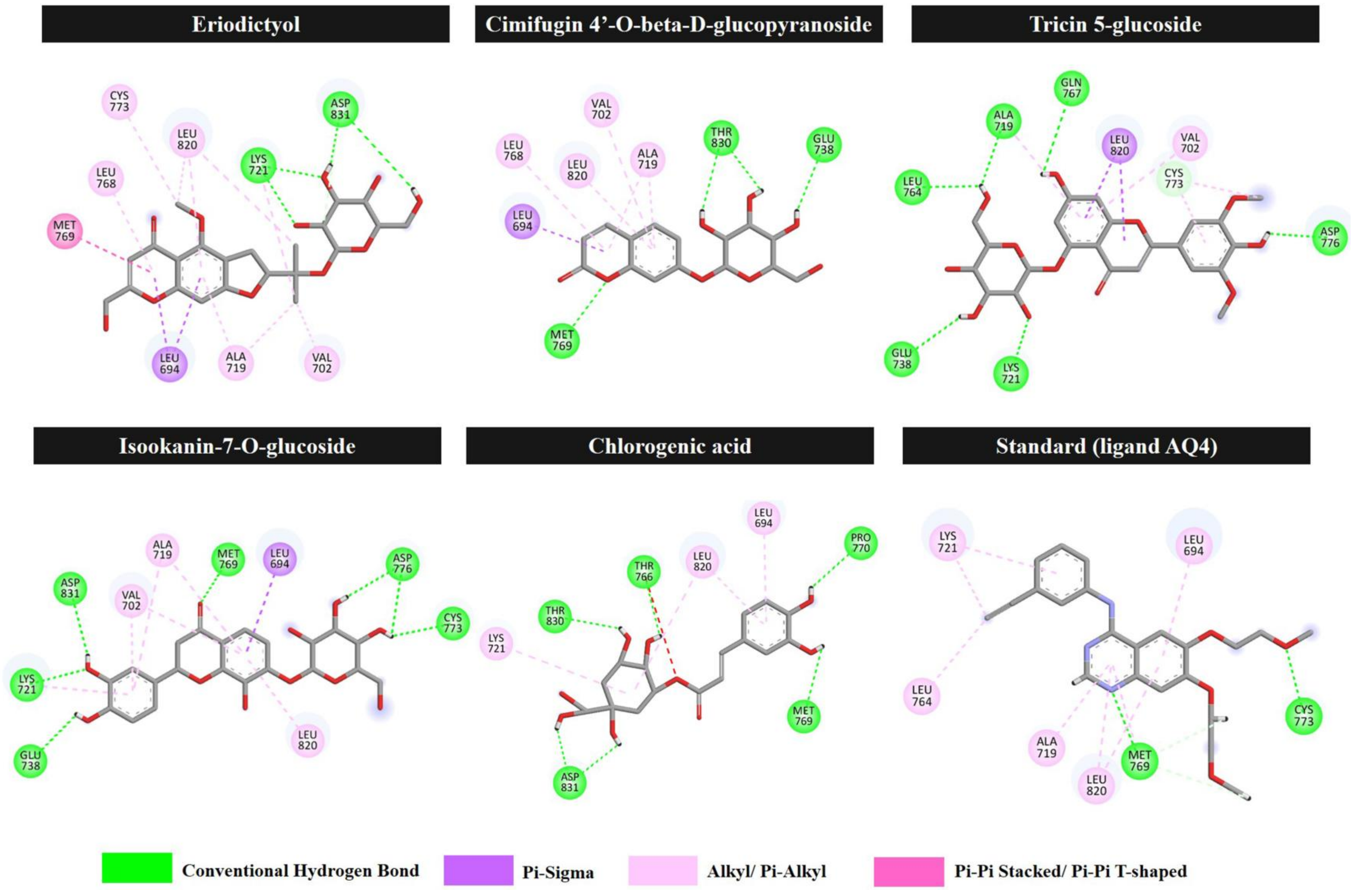

Figure S6. 2D Visualization of molecular binding interactions of compounds with top 5 binding score and co-ligand (standard) against EGFR target.

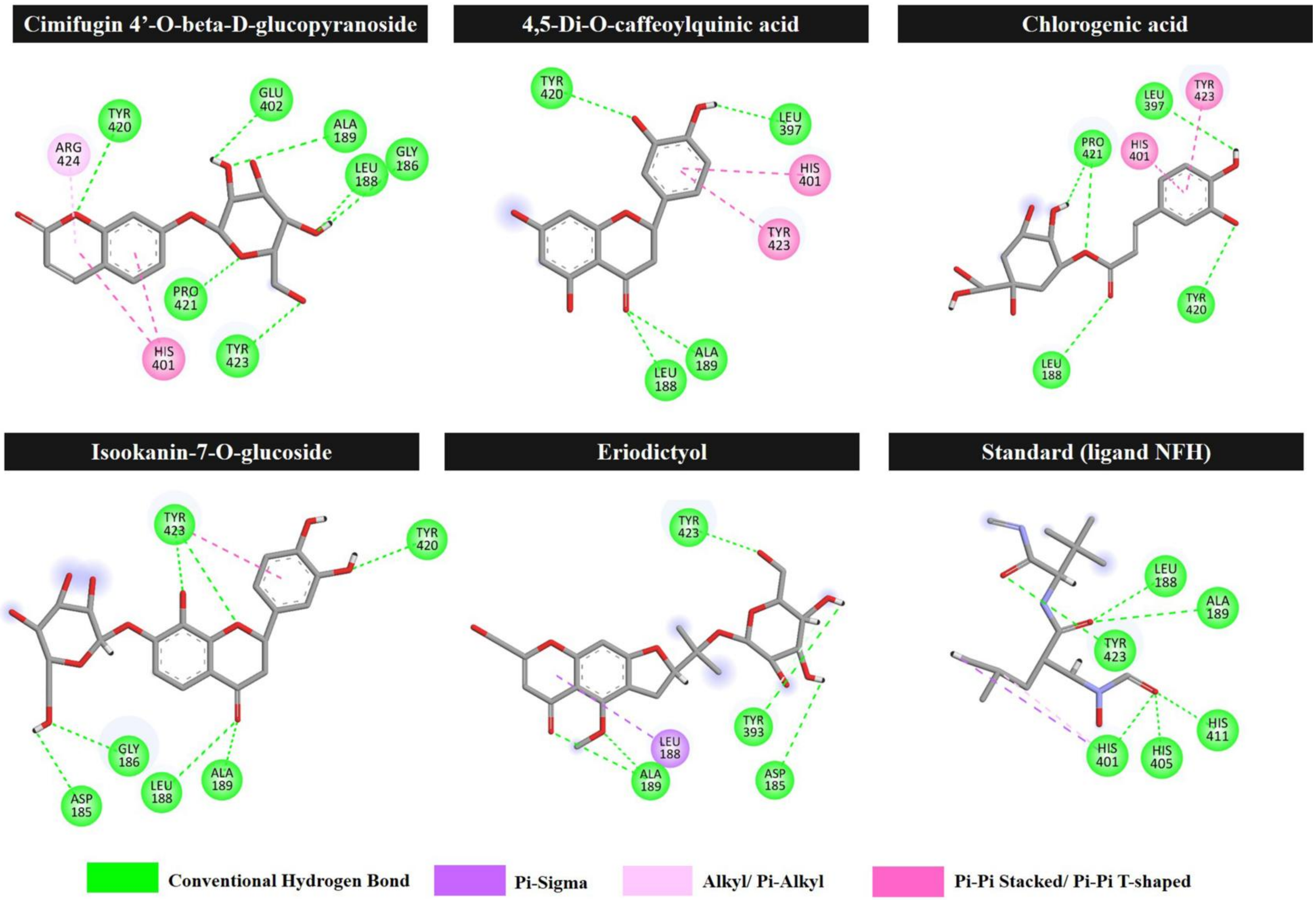

Figure S7. 2D Visualization of molecular binding interactions of compounds with top 5 binding score and co-ligand (standard) against MMP9 target.

Table S1. All compound identification in SS extract under negative electrospray ionization (ESI) mode analyzed by MS-Dial software.

No.	RT (min)	m/z	Mass error (ppm)	Adduct	Compound name	Formula	CAS no.	Intensity	Structure
1	0.657	195.05096	-2.31	$[M-H]^-$	D-Gluconic acid	$C_6H_{12}O_7$	526-95-4	72,882.84	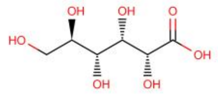
2	1.238	128.03499	0.08	$[M-H]^-$	Pyroglutamic acid	$C_5H_7NO_3$	149-87-1	932,236.97	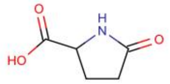
3	2.646	151.02603	-6.89	$[M-H]^-$	Xanthine	$C_5H_4N_4O_2$	69-89-6	908,785.77	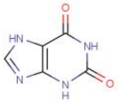
4	2.656	283.06808	-0.64	$[M-H]^-$	Xanthosine	$C_{10}H_{12}N_4O_6$	146-80-5	1,668,782.86	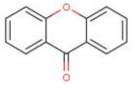
5	2.764	164.07135	-1.1	$[M-H]^-$	L-(-)-Phenylalanine	$C_9H_{11}NO_2$	150-30-1	90,437.55	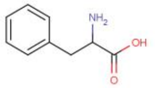
6	3.250	353.08728	1.47	$[M-H]^-$	Chlorogenic acid	$C_{16}H_{18}O_9$	58108-99-9	15,747,212.37	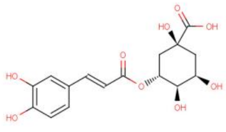
7	3.392	203.08214	2.27	$[M-H]^-$	Tryptophan	$C_{11}H_{12}N_2O_2$	54-12-6	229,920.43	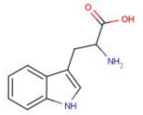
8	3.667	327.07312	-3	$[M-H]^-$	Bergenin	$C_{14}H_{16}O_9$	477-90-7	10,815.82	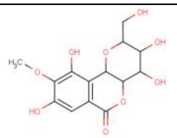

Table S1. All compound identification in SS extract under negative electrospray ionization (ESI) mode analyzed by MS-Dial software (cont.).

No.	RT (min)	m/z	Mass error (ppm)	Adduct	Compound name	Formula	CAS no.	Intensity	Structure
9	3.776	175.0609	-1.31	$[M-H]^-$	2-Isopropylmalic acid	$C_7H_{12}O_5$	3237-44-3	311,339.73	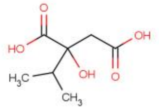
10	3.839	191.05772	-8.43	$[M-H]^-$	D-(-)-quinic acid	$C_7H_{12}O_6$	77-95-2	31,915,474.19	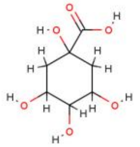
11	3.941	293.12381	-0.41	$[M-H]^-$	Glutamylphenylalanine	$C_{14}H_{18}N_2O_5$	7432-24-8	286,091.60	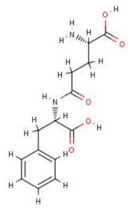
12	4.024	179.03464	-1.06	$[M-H]^-$	Caffeic acid	$C_9H_8O_4$	331-39-5	1,766,512.21	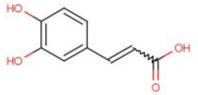
13	4.597	465.14343	-6.11	$[M-H]^-$	Agnuside	$C_{22}H_{26}O_{11}$	11027-63-7	400,844.49	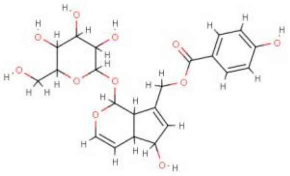
14	4.732	491.11819	2.69	$[M-H]^-$	Tricin 5-glucoside	$C_{23}H_{24}O_{12}$	32769-00-9	2,249,643.12	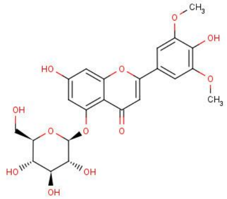
15	5.056	161.02426	0.93	$[M-H]^-$	Umbelliferone	$C_9H_6O_3$	93-35-6	293,225.72	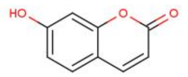

Table S1. All compound identification in SS extract under negative electrospray ionization (ESI) mode analyzed by MS-Dial software (cont.).

No.	RT (min)	m/z	Mass error (ppm)	Adduct	Compound name	Formula	CAS no.	Intensity	Structure
16	5.368	447.09314	0.34	$[M-H]^-$	Plantaginin	$C_{21}H_{20}O_{11}$	26046-94-6	34,018.13	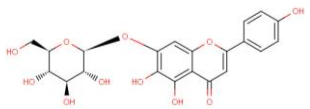
17	5.584	144.04543	0.42	$[M-H]^-$	4-Hydroxyquinoline	C_9H_7NO	529-37-3	128,413.51	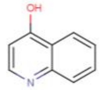
18	5.634	515.12286	-8.74	$[M-H]^-$	Phenylacetic acid	$C_8H_8O_2$	103-82-2	260,847.69	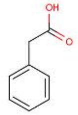
19	5.907	515.12286	-7.11	$[M-H]^-$	3,4-di-O-caffeoylquinic acid	$C_{25}H_{24}O_{12}$	14534-61-3	6,712,534.48	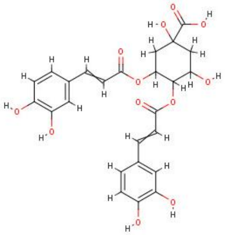
20	5.966	287.05594	0.63	$[M-H]^-$	Eriodictyol	$C_{15}H_{12}O_6$	4049-38-1	166,345.34	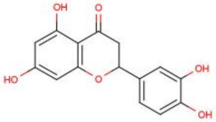
21	5.976	449.1084	1.22	$[M-H]^-$	Isookanin-7-O-glucoside	$C_{21}H_{22}O_{11}$	577-38-8	1,791,031.72	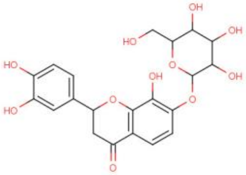
22	6.007	187.09723	1.98	$[M-H]^-$	Azelaic acid	$C_9H_{16}O_4$	123-99-9	312,832.91	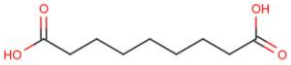

Table S1. All compound identification in SS extract under negative electrospray ionization (ESI) mode analyzed by MS-Dial software (cont.).

No.	RT (min)	m/z	Mass error (ppm)	Adduct	Compound name	Formula	CAS no.	Intensity	Structure
23	12.632	277.21698	4.73	$[M-H]^-$	a-Linolenic acid	$C_{18}H_{30}O_2$	463-40-1	453,463.01	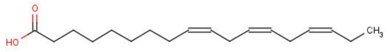
24	14.214	271.23178	-16.41	$[M-H]^-$	16-Hydroxyhexadecanoic acid	$C_{16}H_{32}O_3$	506-13-8	226,579.14	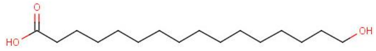
25	14.567	279.23273	2.08	$[M-H]^-$	Linoelaidic acid	$C_{18}H_{32}O_2$	506-21-8	890,990.29	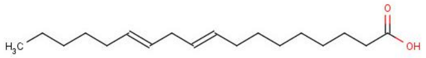

Table S2. All compound identification in SS extract under positive electrospray ionization (ESI) mode analyzed by MS-Dial software.

No.	RT (min)	m/z	Mass error (ppm)	Adduct	Compound name	Formula	CAS no.	Intensity	Structure
1	0.702	138.0577	-15.94	$[M+H]^+$	Trigonelline	$C_7H_7NO_2$	535-83-1	3,833,634.89	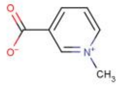
2	0.737	175.1193	-1.83	$[M+H]^+$	Arginine	$C_6H_{14}N_4O_2$	1119-34-2	113,759.63	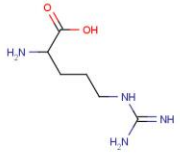
3	0.78	132.1028	-6.59	$[M+H]^+$	Isoleucine	$C_6H_{13}NO_2$	3107-04-8	456,255.99	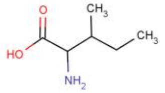
4	0.934	118.0866	-3.13	$[M+H]^+$	Valine	$C_5H_{11}NO_2$	516-06-3	1,106,310.37	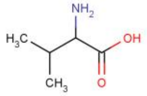
5	0.982	146.0925	4.04	$[M+H]^+$	4-Guanidinobutyric acid	$C_5H_{11}N_3O_2$	463-00-3	116,777.44	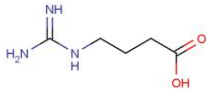
6	1.514	182.0813	2.58	$[M+H]^+$	Tyrosine	$C_{18}H_{32}O_7$	556-03-6	364,690.87	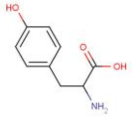
7	2.654	144.0482	-2.22	$[M+H]^+$	4-Methyl-5-thiazoleethanol	C_6H_9NOS	137-00-8	3,493,319.64	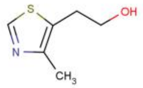
8	2.778	207.0708	-3.82	$[M+H]^+$	7,8-Dimethoxycoumarin	$C_{11}H_{10}O_4$	2445-80-9	28,472,217.11	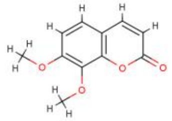
9	2.998	208.069	4.95	$[M+Na]^+$	1,2,3,9-Tetrahydro-4H-carbazol-4-one	$C_{12}H_{11}NO$	15128-52-6	2,097,354.56	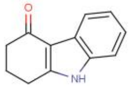

Table S2. All compound identification in SS extract under positive electrospray ionization (ESI) mode analyzed by MS-Dial software (cont.).

No.	RT (min)	m/z	Mass error (ppm)	Adduct	Compound name	Formula	CAS no.	Intensity	Structure
10	3.409	165.0545	3.76	$[M+H]^+$	*p*-Coumaric acid	$C_9H_8O_3$	7400-08-0	348,729.21	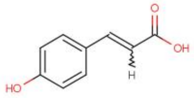
11	3.422	144.0824	-10.9	$[M+H]^+$	2-Naphthylamine	$C_{10}H_9N$	91-59-8	1,939,799.29	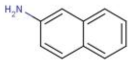
12	3.864	377.0897	0.74	$[M+Na]^+$	Chlorogenic acid	$C_{16}H_{18}O_9$	327-97-9	14,483,893.65	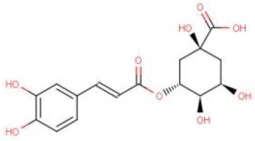
13	3.936	266.1406	-9.17	$[M]^+$	Caffeoylcholine	$C_{14}H_{20}NO_4$	87189-10-4	8,542,735.80	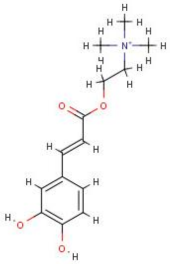
14	3.974	250.145	-6.96	$[M]^+$	4-Coumaroylcholine	$C_{14}H_{20}NO_3$	-	1,891,486.15	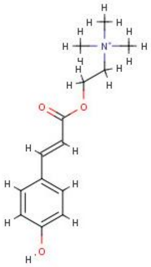

15	5.25	291.0865	12.06	$[M+H]^+$	(+)Catechin	$C_{15}H_{14}O_6$	13392-26-2	101,864.12	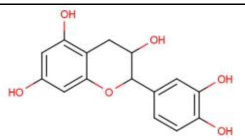

Table S2. All compound identification in SS extract under positive electrospray ionization (ESI) mode analyzed by MS-Dial software (cont.).

No.	RT (min)	m/z	Mass error (ppm)	Adduct	Compound name	Formula	CAS no.	Intensity	Structure
16	5.307	197.1075	-0.71	$[M+H]^+$	1-Ethyl-9H-pyrido[3,4-b]indole	$C_{13}H_{12}N_2$	20127-61-1	351,545.59	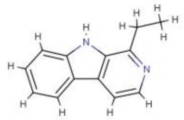
17	5.503	247.1078	1.17	$[M+H]^+$	N-Acetyltryptophan	$C_{13}H_{14}N_2O_3$	87-32-1	260,980.33	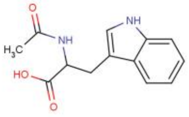
18	5.925	539.1169	5.79	$[M+Na]^+$	3,4-Di-O-caffeoylquinic acid	$C_{25}H_{24}O_{12}$	14534-61-3	1,047,633.00	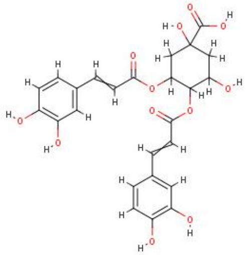
19	5.99	289.0712	-4.22	$[M+H]^+$	Eriodictyol	$C_{15}H_{12}O_6$	4049-38-1	957,710.88	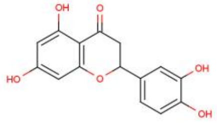
20	6.159	491.1538	-7.78	$[M+Na]^+$	Cimifugin 4'-O-beta-D-glucopyranoside	$C_{22}H_{28}O_{11}$	1632110-81-6	1,701,413.79	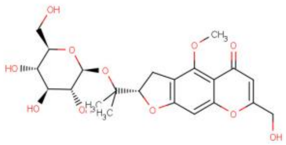

21	6.453	325.092	-0.74	[M+H]+	Skimmin	$C_{15}H_{16}O_8$	93-39-0	1,086,358.43	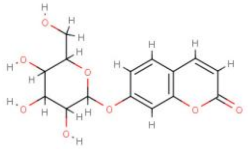

Table S2. All compound identification in SS extract under positive electrospray ionization (ESI) mode analyzed by MS-Dial software (cont.).

No.	RT (min)	m/z	Mass error (ppm)	Adduct	Compound name	Formula	CAS no.	Intensity	Structure
22	8.426	543.1837	-6.74	[M+Na]+	Pinoresinol 4-O-glucoside	$C_{26}H_{32}O_{11}$	69251-96-3	233,327.64	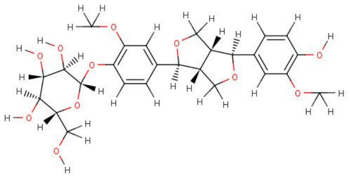
23	12.454	184.0734	2.01	[M+H]+	Phosphocholine	$C_5H_{15}NO_4P$	645-84-1	1,755,807.03	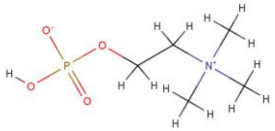

Table S3. Binding interactions of 21 compounds form the SS extract corresponding NF-κB, EGFR, and MMP9 proteins involved in a wound healing.

Compound names	NF-κB			EGFR			MMP9		
	**Inhibition constant, *K*i (μM)**	**Hydrogen bonds (Å)**	**Hydrophobic interactions (Å)**	**Inhibition constant, *K*i (μM)**	**Hydrogen bonds (Å)**	**Hydrophobic interactions (Å)**	**Inhibition constant, *K*i (μM)**	**Hydrogen bonds (Å)**	**Hydrophobic interactions (Å)**
Pyroglutamic acid	654.52	CYS99(2.48, 2.04), GLU97(1.79)	-	2200	MET769(1.95), GLN767(2.09)	-	1400	ALA189(2.01, 1.85), HIS401(2.12), HIS405(2.40), HIS411(1.95), GLU402(2.01)	-
Xanthine	450.90	LYS44(1.73)	ASP166(2.16), MET65(4.87), VAL29(5.01), LYS44(5.05), MET96(5.49), ILE165(4.08), LYS44(5.42), MET96(4.74), ILE165(4.46)	1330	MET769(2.04), GLN767(1.72)	THR766(2.93), LEU768(2.66), ALA719(3.85, 5.09), CYS751(5.49), MET769(5.34), LEU820(4.55, 4.94)	162.64	ALA189(3.08, 2.52), HIS401(1.78), HIS405(2.28), HIS411(1.71), PRO421(2.85, 2.55), GLU402(2.10)	-
Xanthosine	41.27	ASP103(2.74), LEU21(2.09), GLU97(2.02, 2.00)	LEU21(2.60), TYR98(5.63), LEU21(4.92), VAL152(3.72, 4.89)	51.98	LYS721(2.10), MET769(1.86), GLN767(1.98), THR766(2.32), ASP831(2.18, 1.89)	THR766(3.07), LEU768(2.81), VAL702(4.63), ALA719(3.95, 3.80), LEU820(5.01, 4.44), MET769(5.12)	22.64	LEU188(1.74), ALA189(2.61, 3.07), HIS401(2.10), HIS405(2.46), HIS411(1.81, 2.32), GLU402(2.12)	-
D-(-)-Quinic acid	1740	LEU21(2.95, 2.05), CYS99(2.13, 2.74)	-	1520	THR766(2.02), ASP831(2.03), GLU738(2.00)	-	1610	ALA189(2.40, 2.51), TYR423(1.76),	-

								GLY186(1.91)	
Caffeic acid	122.65	GLU97(1.95), ASP103(1.80, 1.92),	LEU21(4.58), VAL152(5.15)	227.20	GLN767(2.02), THR830(1.73), GLU738(2.12)	ALA719(25.33), LYS721(4.57)	145.09	LEU188(2.16), ALA189(2.62), ARG424(2.03), GLU402(1.77)	HIS401(5.19)

Å: Binding in Angstrom. Co-ligands of NF-κB (ligand KSA), EGFR (ligand AQ4) and MMP9 (ligand NFH) targets.

Table S3. Binding interactions of 21 compounds form the SS extract corresponding NF-κB, EGFR, and MMP9 proteins involved in a wound healing (cont.).

Compound names	NF-κB			EGFR			MMP9		
	**Inhibition constant, *K*i (μM)**	**Hydrogen bonds (Å)**	**Hydrophobic interactions (Å)**	**Inhibition constant, *K*i (μM)**	**Hydrogen bonds (Å)**	**Hydrophobic interactions (Å)**	**Inhibition constant, *K*i (μM)**	**Hydrogen bonds (Å)**	**Hydrophobic interactions (Å)**
Tricin 5-glucoside	0.02716	CYS99(1.92, 2.34), LYS106(2.80), ASP166(2.03), LEU21(2.28), ASP103(2.01), GLU97(2.03), ASP166(1.88)	LYS44(4.65, 5.17), LEU94(5.15), MET96(4.63), VAL29(4.76, 4.41, 4.47), LYS44(4.76), LEU21(5.04), VAL152(4.86, 4.54), ILE165(4.82, 4.61), ALA42(4.38), CYS99(5.37)	8.31	LYS721(2.53), ALA719(2.09), LEU764(2.06), GLU738(2.41), GLN767(2.82), ASP776(1.71)	LEU820(3.68, 3.55), CYS773(3.92, 3.96), VAL702(5.37), ALA719(4.49)	7.81	ALA191(3.04), GLU111(3.01), GLU402(2.20, 1.87)	TYR423(2.79), TYR393(5.36), TYR423(4.73)
3,4-Di-O-caffeoylquinic acid	0.19143	THR23(1.62), GLU97(2.00, 1.74), CYS99(2.77, 1.92), GLU149(1.90), THR23(2.48, 2.01)	VAL29(4.93), ALA42(4.01), MET96(5.34), VAL152(4.74), ILE165(4.74)	19.37	GLU738(2.27), MET769(1.84), ASP831(1.80, 1.98)	LYS721(2.63), LS721(2.98), PHE699(5.49), VAL702(4.87), LEU694(3.97), ALA719(5.47)	6.45	GLY186(2.27), LEU188(1.86), ALA189(2.36), HIS411(2.71), PRO421(2.58), TYR393(2.18, 2.00), ASP185(2.77)	HIS401(4.00), TYR393(5.30), HIS411(5.42)
Isookanin-7-O-glucoside	0.04243	ASP166(2.10, 1.86), GLU97(1.80, 2.09)	VAL152(3.79), VAL29(5.27), LEU21(5.45), ALA42(4.57), CYS99(4.87), ILE165(4.90)	9.95	LYS721(2.17), MET769(1.77), ASP776(2.14, 2.17), CYS773(2.55), GLU738(2.02), ASP831(2.27)	LEU694(3.58), VAL702(5.14, 5.36), ALA719(5.08, 5.43), LEU820(5.43), LYS721(4.21	0.45899	TYR420(2.18), TYR423(2.98, 1.66), ASP185(2.10)	TYR423(5.49)
Linoelaidic acid	8.75	LYS44(1.76), ASP166(2.00,	LEU21(5.03, 4.92, 4.92), VAL29(3.32,	78.48	-	LEU768(2.65), VAL702(3.87, 4.10,	74.21	TYR423(2.08, 1.99)	HIS401(3.55), HIS401(4.67),

		2.14)	4.75), VAL152(5.12, 3.88, 4.48), ALA42(3.91, 4.48), MET96(4.12), ILE165(4.12), CYS99(4.68, 4.27), TYR98(5.26), VAL74(5.20, 5.07)			4.35), ALA719(4.85), LYS721(4.27, 3.99), LEU694(4.72, 4.90), LEU764(4.39), LEU820(5.23)			TYR423(4.05)

Å: Binding in Angstrom. Co-ligands of NF-κB (ligand KSA), EGFR (ligand AQ4) and MMP9 (ligand NFH) targets.

Table S3. Binding interactions of 21 compounds form the SS extract corresponding NF-κB, EGFR, and MMP9 proteins involved in a wound healing (cont.).

**Compound names**	**NF-κB**			**EGFR**			**MMP9**		
	**Inhibition constant, *K*i (μM)**	**Hydrogen bonds (Å)**	**Hydrophobic interactions (Å)**	**Inhibition constant, *K*i (μM)**	**Hydrogen bonds (Å)**	**Hydrophobic interactions (Å)**	**Inhibition constant, *K*i (μM)**	**Hydrogen bonds (Å)**	**Hydrophobic interactions (Å)**
Trigonelline	822.00		LEU21(4.33), TYR98(5.46)	1110	LYS704(2.05)	LEU694(4.39)	1410	-	TYR423(3.14), HIS401(3.88)
Valine	1100	LYS106(1.80), ASP103(1.94, 2.03), LEU21(2.30)	-	796.13	ASP831(1.80, 1.86, 1.82)	VAL702(4.78)	391.42	HIS401(1.94), HIS405(1.92), HIS411(1.84), GLU402(2.41, 1.81)	HIS401(4.25)
4-Methyl-5-thiazoleethanol	772.57	CYS99(2.94, 1.82), GLU97(1.98)	VAL152(5.11, 4.53), LEU21(4.36, 4.06)	673.95	GLU738(1.96)	THR766(3.87), MET742(5.49), ALA719(3.34, 4.66), VAL702(4.01), LYS721(4.78, 4.08)	158.69	ARG424(2.07)	TYR423(5.31), HIS401(3.96), TYR423(5.58), HIS401(4.95)
7,8-Dimethoxycoumarin	46.14	CYS99(1.92), ASP103(2.28)	LEU21(4.72, 4.32, 4.33), VAL152(4.55, 4.37, 4.50), CYS99(5.15)	129.88	-	LEU768(2.24), PRO770(2.67, 3.03), LEU694(2.70), LEU694(4.10, 5.23), LYS704(4.56), LEU768(4.13, 5.48), ALA719(4.34), LEU820(4.91)	17.63	LEU188(2.14), PRO421(3.07), ARG424(2.91)	TYR423(5.46)
1,2,3,9-Tetrahydro-4H-	9.77	CYS99(1.67)	VAL29(5.23), VAL74(5.09), VAL152(4.47, 4.17,	60.89	MET769(1.78)	LEU768(2.44), VAL702(4.61), LEU820(4.61),	1.09	-	GLU402(4.14), HIS401(3.82, 4.14), HIS401(5.12)

carbazol-4-one			4.93), ILE165(3.96), LEU21(4.36, 4.12)			LEU694(4.23, 4.08)			
2-Naphthylamine	101.31	LEU21(2.13), ASP103(2.15)	LEU21(3.94, 3.91), VAL152(3.82), ALA42(5.04), CYS99(5.07), VAL152(5.08)	113.19	THR830(1.94), ASP831(1.98)	LYS721(4.33), ALA719(4.64), LYS721(4.38, 3.83), LEU764(5.37)	22.16	GLU402(2.12)	HIS401(3.86, 5.36), TYR423(5.09)

Å: Binding in Angstrom. Co-ligands of NF-κB (ligand KSA), EGFR (ligand AQ4) and MMP9 (ligand NFH) targets.

Table S3. Binding interactions of 21 compounds form the SS extract corresponding NF-κB, EGFR, and MMP9 proteins involved in a wound healing (cont.).

Compound names	NF-κB			EGFR			MMP9		
	**Inhibition constant, *K*i (μM)**	**Hydrogen bonds (Å)**	**Hydrophobic interactions (Å)**	**Inhibition constant, *K*i (μM)**	**Hydrogen bonds (Å)**	**Hydrophobic interactions (Å)**	**Inhibition constant, *K*i (μM)**	**Hydrogen bonds (Å)**	**Hydrophobic interactions (Å)**
Chlorogenic acid	1.12	LYS44(2.02), CYS99(2.06, 2.48), ASP166(2.16), GLU97(1.82), ASP166(2.02), GLU149(1.98, 1.83)	VAL152(3.99), VAL29(4.86), ILE165(4.99, 5.43), LEU21(4.77), ALA42(4.41), CYS99(5.21)	11.69	MET769(2.36), PRO770(2.86), THR766(2.05), ASP831(1.76, 1.73), THR830(2.19)	LEU694(3.81), LYS721(5.27), LEU820(5.47, 5.38)	0.29992	LEU188(2.10), TYR420(2.84), PRO421(2.74, 2.00), LEU397(1.93)	HIS401(4.05), TYR423(5.41)
Caffeoylcholine	21.52	ASP103(4.00), CYS99(2.01), ASP103(2.49), GLU97(1.71, 1.90)	VAL152(3.97), ALA42(4.39), ILE165(5.12)	87.24	LYS721(1.81), ASP831(2.15), GLU738(2.12)	MET742(5.62), LYS721(5.16)	1.76	GLU402(3.71), TYR420(2.88), LEU397(1.97)	HIS401(3.98), TYR423(5.47)
4-Coumaroylcholine	28.94	ASP166(4.28), ASP166(2.15), CYS99(2.36)	CYS99(4.87), ALA42(3.95), VAL74(5.20), VAL152(4.41), ILE165(5.28)	75.72	LYS721(1.77), MET769(2.00), GLU738(2.17)	ASP831(4.92), MET742(5.67), LYS721(5.13)	0.95999	GLU402(4.13), LEU188(2.14), ALA189(2.17)	TYR423(5.48)
Eriodictyol	0.41327	CYS99(1.91, 2.61), ASP166(1.77), THR23(2.53), GLU149(1.91), LEU21(2.14)	VAL29(4.39, 3.97, 4.84), ALA42(4.96, 4.88), MET96(5.23), VAL152(5.14, 4.34, 4.82, 4.55), ILE165(3.98, 3.68, 5.30, 5.19), MET96(4.25, 4.25), LYS44(4.79), VAL74(3.61),	6.08	LYS721(1.86, 1.82), ASP831(1.77, 2.70)	LEU694(3.56, 3.89), ALA719(4.47, 4.92), LEU820(4.68, 4.87, 5.00), VAL702(4.81), LYS721(4.51), CYS773(4.29), LEU768(5.24)	0.84371	ALA189(2.78, 2.60, 2.01), TYR423(1.82), ASP185(2.34), TYR393(1.94)	LEU188(2.96)

			CYS99(3.57), LEU21(3.69, 4.58)						

Å: Binding in Angstrom. Co-ligands of NF-κB (ligand KSA), EGFR (ligand AQ4) and MMP9 (ligand NFH) targets.

Table S3. Binding interactions of 21 compounds form the SS extract corresponding NF-κB, EGFR, and MMP9 proteins involved in a wound healing (cont.).

Compound names	NF-κB			EGFR			MMP9		
	Inhibition constant, *K*i (μM)	Hydrogen bonds (Å)	Hydrophobic interactions (Å)	Inhibition constant, *K*i (μM)	Hydrogen bonds (Å)	Hydrophobic interactions (Å)	Inhibition constant, *K*i (μM)	Hydrogen bonds (Å)	Hydrophobic interactions (Å)
Cimifugin 4'-O-beta-D-glucopyranoside	3.56	LYS44(2.05), CYS99(1.99, 2.23), GLU97(1.97, 1.76)	VAL29(4.00, 4.72), ILE165(5.27), LYS44(5.45), ILE165(5.28)	6.05	MET769(2.31), THR830(2.12, 1.79), GLU738(2.09)	PRO770(2.69), VAL702(5.35, 5.14), ALA719(4.15), LEU820(4.57), LEU694(4.48), LEU768(5.29)	0.12691	LEU188(1.79), ALA189(2.35), TYR420(1.92), PRO421(2.87), TYP423(2.13), GLU402(2.01), GLY186(2.04)	HIS401(3.91, 5.36), ARG424(4.67)
Skimmin	3810	ASP103(4.98), CYS99(4.00), GLU97(1.77)	-	9440	ME769(1.77), GLN767(1.96)	-	1160	GLU402(4.37), LEU418(2.97), MET422(2.14)	HIS401(4.31), TYR423(3.76)
Co-ligands	0.0019	CYS99(1.77), GLU149(2.20), GLU97(1.72)	MET96(5.45, 5.78, 4.72), VAL29(4.04, 4.65, 5.50, 4.76), ILE165(3.95, 4.52, 3.99), LEU21(5.31, 4.23, 3.98), ALA42(4.76), VAL152(4.60, 4.45, 4.93), LYS44(5.20)	9.82	MET769(1.86), CYS773(1.97)	LEU764(2.87, 4.54), LYS721(4.22, 4.51), ALA719(3.64,5.36) MET769(5.41), LEU820(4.60, 4.75), LEU694(5.10)	1.71	LEU188(1.87), ALA189(2.50, 2.74), HIS401(1.78), HIS405(2.26), HIS411(1.69), TYR423(1.91)	HIS401(2.64), HIS401(4.28)

Å: Binding in Angstrom. Co-ligands of NF-κB (ligand KSA), EGFR (ligand AQ4) and MMP9 (ligand NFH) targets.

Table S4. The selected ADMET parameters for the compounds obtained from pkCSM online tool.

Parameters	Compounds																				
	**1**	**2**	**3**	**4**	**5**	**6**	**7**	**8**	**9**	**10**	**11**	**12**	**13**	**14**	**15**	**16**	**17**	**18**	**19**	**20**	**21**
**Absorption (A)**																					
Water solubility (log mol/L)	0.103	-3.177	-2.399	-0.047	-1.748	-3.334	-3.016	-3.146	-5.862	-2.019	-2.888	-0.210	-2.546	-2.619	-2.752	-2.321	-1.996	-1.905	-3.144	-1.992	-2.533
Caco-2 permeability (log Papp in 10^{-6} cm/s)	0.567	-0.305	0.192	-0.510	0.114	-0.519	-0.869	0.573	1.570	1.124	0.541	1.762	1.289	1.590	1.484	-0.394	0.280	1.218	0.557	-0.617	0.625
Skin permeability (log Kp)	-3.429	-2.735	-2.735	-3.114	-2.648	-2.735	-2.735	-2.735	-2.723	-2.741	-2.736	-2.890	-2.436	-2.053	-2.591	-2.737	-2.948	-2.851	-2.739	-2.739	-2.742
**Distribution (D)**																					
VDss (log L/kg)	-0.802	-0.387	-0.020	-0.650	-0.595	0.153	0.560	0.358	-0.587	-0.713	-0.572	-0.230	-0.160	0.517	0.444	-0.377	0.871	0.775	0.063	0.171	-0.610
Fraction unbound (Fu)	0.805	0.534	0.914	0.788	0.426	0.145	0.143	0.195	0.054	0.836	0.462	0.623	0.358	0.312	0.145	0.461	0.575	0.474	0.161	0.553	0.394
**Metabolism (M)**																					
CYP2D6 substrate	No	No	No	No	No	No	No	No	No	No	No	No	No	No	No	No	No	No	No	No	No
CYP3A4 substrate	No	No	No	No	No	No	No	No	Yes	No	No	No	No	No	No	No	No	No	No	No	No
CYP1A2 inhibitor	No	No	No	No	No	No	No	No	Yes	No	No	No	Yes	Yes	Yes	No	No	Yes	No	No	No
CYP2C19 inhibitor	No	No	No	No	No	No	No	No	No	No	No	No	No	No	No	No	No	No	No	No	No
CYP2C9 inhibitor	No	No	No	No	No	No	No	No	No	No	No	No	No	No	No	No	No	No	No	No	No
CYP2D6 inhibitor	No	No	No	No	No	No	No	No	No	No	No	No	No	No	No	No	No	No	No	No	No
CYP3A4 inhibitor	No	No	No	No	No	No	No	No	No	No	No	No	No	No	No	No	No	No	No	No	No
**Excretion (E)**																					
Total clearance (log ml/min/kg)	0.605	0.647	0.594	0.646	0.558	0.725	0.057	-0.002	1.936	0.349	0.205	0.449	0.824	0.419	0.228	0.378	0.889	0.881	0.039	0.287	0.783
**Toxicity (T)**																					

AMES toxicity	No	No	No	No	No	Yes	No	Yes	No	No	No	No	No	Yes	Yes	No	No	No	Yes	No	No
Max. tolerated dose (log mg/kg/day)	1.328	0.743	0.198	2.137	0.827	0.852	0.85	0.962	-0.827	0.68	1.137	1.087	0.977	-0.211	0.526	0.621	-0.375	0.208	0.405	0.173	0.663
Skin sensitisation	No	No	No	No	No	No	No	No	Yes	No	No	Yes	No	No	Yes	No	No	No	No	No	No
